# The Influence of Clinically Diagnosed Neuropathy on Respiratory Muscle Strength in Type 2 Diabetes Mellitus

**DOI:** 10.1155/2018/8065938

**Published:** 2018-11-29

**Authors:** Birgit L. M. Van Eetvelde, Dirk Cambier, Karsten Vanden Wyngaert, Bert Celie, Patrick Calders

**Affiliations:** Department of Rehabilitation Sciences and Physiotherapy, Ghent University, Ghent, Belgium

## Abstract

**Objectives:**

This cross-sectional study investigated the influence of clinically diagnosed neuropathy (cdNP) on respiratory muscle strength in patients with type 2 diabetes mellitus (T2DM).

**Methods:**

110 T2DM patients and 35 nondiabetic healthy controls (≥60 years) were allocated to one of three groups depending on the presence of cdNP: T2DM without cdNP (D−; *n* = 28), T2DM with cdNP (D+; *n* = 82), and controls without cdNP (C; *n* = 35). Clinical neurological diagnostic examination consisted of Vibration Perception Threshold and Diabetic Neuropathy Symptom score. Respiratory muscle strength was registered by maximal Inspiratory and Expiratory Pressures (PI_max_ and PE_max_), and respiratory function by Peak Expiratory Flow (PEF). Isometric Handgrip Strength and Short Physical Performance Battery were used to evaluate peripheral skeletal muscle strength and physical performance. Univariate analysis of covariance was used with age, level of physical activity, and body mass index as covariates.

**Results:**

PI_max_, PE_max_, and PEF were higher in C compared to D− and D+. Exploring more in detail, PI_max_, PE_max_, and PEF were significantly lower in D+ compared to C. PE_max_ and PEF were also significantly lower in D− versus C. Measures of peripheral muscle strength and physical performance showed less associations with cdNP and T2DM.

**Conclusions:**

The presence of cdNP affects respiratory muscle strength in T2DM patients compared to healthy controls. Both cdNP and diabetes in themselves showed a distinctive impact on respiratory muscle strength and function; however, an accumulating effect could not be ascertained in this study. As commonly used measures of peripheral muscle strength and physical performance seemed to be less affected at the given time, the integration of PI_max_, PE_max_, and PEF measurements in the assessment of respiratory muscle weakness could be of added value in the (early) screening for neuropathy in patients with T2DM.

## 1. Introduction

Type 2 diabetes mellitus (T2DM) is the most common cause of (sensori) motor and autonomic neuropathy [1, 2]. One of the most important and well-recognized clinical manifestations of diabetes-associated neuropathy (NP) is impairment and debilitation in functioning and locomotion due to the development of lower limb skeletal muscle weakness, which is closely related to the severity of NP [3, 4]. Studies using magnetic resonance imaging (MRI) showed accelerated muscle atrophy in accordance with an increased loss of muscle strength in patients with T2DM suffering from symptomatic NP in comparison to T2DM patients without NP and healthy controls [5, 6].

For that matter and from a clinical point of view, the assessment of the influence of NP on muscle function is highly recommended and mainly achieved by means of standardized clinical examinations such as manual muscle testing, isometric and isokinetic dynamometry, Handgrip Strength (HGS), and by functional performance tools (e.g., timed chair stand test (CST) and indirectly by gait analysis and appraisal) [5, 7–9].

Approximately 10–15% of all people aged >40 suffers from NP in which diabetes remains the most common cause. Asides age, diabetes and a set of other distinctive factors causing NP has to be classified as idiopathic in 20–30% of all patients suffering from NP even after thorough investigation. This idiopathic NP is considered as a major culprit of a person's disability with important social impact due to pain, gait instability, increased risk of falls, injuries, and poor quality of life [10–12].

The association between reduced respiratory function and T2DM has already been described [13], however, the underlying mechanisms are still undisclosed. Klein et al. reported in a systematic review an inverse association between respiratory function on the one hand and blood glucose levels, the severity and duration of T2DM on the other, independent of smoking status or presence of obesity [14]. van den Borst et al. also reported a decreased lung function in T2DM. Metaregression analysis showed, however, that this relation could not be explained by body mass index (BMI), smoking, diabetes duration, or glycated hemoglobin (HbA1c) [15]. Respiratory muscle strength is strongly associated with pulmonary function and may play important roles in the respiratory network, which on its turn depends on intact neural circuitry that orchestrates the interplay between respiratory muscles and intrinsic pulmonary function to maintain adequate ventilation [16]. Kabitz et al. showed that impaired respiratory neuromuscular function, which is strongly related to diabetic polyneuropathy, occurs in T2DM patients as assessed by nonvolitional gold-standard phrenic nerve stimulation [4]. Also, other studies have reported that in patients with T2DM, respiratory muscle weakness can occur and might be associated with autonomic dysfunction [17, 18]. In contrast to the large number of studies examining peripheral muscle weakness in T2DM patients with NP [3, 5, 8, 19–22], only limited research has been conducted regarding the impact of diabetic—or any kind for that matter—NP on respiratory muscle strength [4].

The aim of the present study is to evaluate respiratory muscle strength and function in T2DM patients with clinically diagnosed neuropathy (cdNP) and to compare this with T2DM patients without cdNP and healthy controls. We hypothesize that, compared to healthy controls, respiratory muscle strength and function are decreased in T2DM patients without cdNP and even more impaired in the presence of cdNP.

With respect to the aforementioned hypothesis, the assessment of maximal static Inspiratory and Expiratory Pressure measurements and Peak Expiratory Flow could be considered regarding its added value in the screening for NP.

## 2. Materials and Methods

### 2.1. Study Design and Population

In this cross-sectional case-control study, 110 patients with T2DM and 35 healthy controls were included (*n* = 145).

Participants comprised both community-dwelling elderly and elderly living in a residential care setting. Patients with T2DM were recruited by the Department of Endocrinology (University Hospital Ghent) and their general practitioner, and healthy controls by online advertising and flyer distribution. T2DM was diagnosed on two different occasions based on HbA1c assessments according to the Type 2 Diabetes ADA Diagnosis Criteria [23].

Criteria for inclusion were (i) aged 60 years or more, (ii) living in the community or residential care setting, (iii) able to respond adequately to Dutch instructions, and (iv) able to walk independently with or without walking aids. Subjects suffering from (i) major neurological conditions (e.g., stroke, Parkinson's disease, and dementia), (ii) musculoskeletal disabilities (e.g., foot ulcerations, lower extremity amputations, and arthritis with limited joint mobility precluding ambulation), (iii) severe cardiovascular disorders (e.g., exercise-induced chest pain, congestive heart failure (New York Heart Association class III and IV)), and (iv) respiratory diseases (e.g., exercise-induced asthma and COPD (Global Initiative for Chronic Obstructive Lung Diseases (GOLD)) stages 3 and 4) were excluded.

Based on a clinical neurological diagnostic examination performed by trained physical therapists, the population could be divided into three groups: T2DM without cdNP (D−; *n* = 28), T2DM with cdNP (D+; *n* = 82), and nondiabetic healthy controls without cdNP (C; *n* = 35). Control subjects having NP after the clinical neurological examination were excluded as the differentiating etiology of NP was not further examined.

The Ethical Committee of the Ghent University Hospital gave approval to this study, and all participants signed an informed consent. Consequently, this research is compliant with the World Medical Association Declaration of Helsinki – Ethical Principles for Medical Research Involving Human Subjects.

### 2.2. Outcome Measurements

All measurements were performed on a single morning in a quiet setting and well-lit room with flat surface. At first, anthropometrical data and HbA1c were obtained followed by NP-examinations. Subsequently, respiratory muscle strength, HGS, and the Short Physical Performance Battery (SPPB) were assessed, and a physical activity questionnaire was completed.

### 2.3. Patient Characteristics

Height, weight, BMI, and body composition (bioelectrical impedance analysis; BIA, Bodystat® 1500MDD) were measured and calculated.

HbA1c was measured using the A1CNow SELFCHECK® (Bayer), an instrument which is well correlated with standardized laboratory HbA1c tests (*r* = 0.758) [24].

Habitual physical activity levels were measured using the physical activity questionnaire for the elderly [25, 26].

### 2.4. Measurements of Peripheral Clinically Diagnosed Neuropathy

The clinical neurological diagnostic examination consisted of two parts: measurements of the Vibration Perception Threshold (VPT), an assessment of the peripheral large-fiber sensory nerve function, and the Diabetic Neuropathy Symptom score questionnaire (DNS).

The VPT, a valid and reliable measurement, was determined using a Bio-Thesiometer® (Bio Medical Instrument Co., Ohio, USA) on the left and right medial malleolus and on the distal plantar surface of the big toes [27, 28]. VPT was defined as the lowest recorded voltage when subjects indicated the sense of vibration. Each measurement was repeated three times and the lowest reading was considered [29, 30]. Since the threshold at which vibration becomes perceptible is dependent of age, gender, and location, four percentile rank charts of VPT variation were used [29]. To decide whether vibration perception was within the normal range, a normality cut-off on the 95th percentile was applied. If one of the readings (big toe and the medial malleolus, both left and right) was above the 95th percentile, this criterion was classified as positive.

The DNS, a validated 4-point yes/no questionnaire, has a high predictive value in the screening for diabetic NP when patients score ≥1/4. Meijer et al. compared the validity, predictive value, and reproducibility of the DNS with the Neuropathy Symptom Score (NSS). They found a high correlation between NSS and DNS score (*r* = 0.88) and concluded that the DNS is a fast, easy, reproducible (Cohen's weighted kappa 0.78–0.95), and valid assessment tool to screen for diabetic polyneuropathy [31].

Patients with T2DM were classified as having peripheral cdNP based on at least one of two positive criteria: a VPT exceeding the normality cut-off of 95% or a DNS score of ≥1/4.

### 2.5. Measurements of Muscle Strength

The maximal static Inspiratory and Expiratory Pressure measurements (PI_max_ and PE_max_; cm H_2_O) were obtained by a Pocket-Spiro Mouth Pressure Monitor with a differential pressure transducer (MPM100; Medical Electronic Construction®). To measure PI_max_, subjects were seated and asked to exhale slowly and completely up to residual volume and then to perform a maximum inspiratory maneuver during at least 1.5 seconds against a completely occluded airway. Then, a 1-second average including the peak pressure was calculated, indicating the inspiratory muscle strength. PE_max_ was determined under the same conditions while first inhaling completely up to total lung capacity and then performing a maximum expiratory maneuver. For each index, three tests were recorded, and the highest value was used for data analysis [32–35]. The measurement of the maximum static mouth pressures produced against an occluded airway is the most widely used method of measurement and is an easy way to gauge respiratory muscle strength and to determine the severity of respiratory muscle strength impairments [36]. Additionally, Peak Expiratory Flow (PEF; L/m), a cheap, simple, and widely accessible technique with a prognostic value for morbidity [37, 38], was recorded using a Mini-Wright Peak Flow Meter (Henrotech®). This is internationally recognized as the golden standard for PEF measurements [39]. PEF is used as an indicator for respiratory muscles strength in subjects without lung disorders [37].

Isometric HGS (kg) was measured according to the American Society of Hand Therapists guidelines [40] using the Jamar® dynamometer (Sammons Preston Rolyan Inc., Bolingbrook, IL) at the dominant side [37]. The highest grip score of three consecutive trials was retained.

### 2.6. Measurements of Physical Performance

The SPPB consists of a timed standing balance test (feet together side-by-side, semitandem, and tandem stance), a walk test (time to walk 2.44 meters at usual pace), and a CST (time to raise from a chair and return to the seated position in five times) [41]. Each of the three component tasks was rated from 0 (unable to complete) to 4 (best), and a compiled score was computed by the sum of scores on component tasks (range 0 = worst to 12 = best) [42, 43]. This composite test is often used and validated as a standard assessment of physical performance in research and clinical practice of the ageing population [37, 44].

### 2.7. Statistical Analysis

Data were analyzed using IBM Statistical Package for Social Sciences (SPSS version 24 for Windows) and were considered significant at *α* < 0.05. After confirming the approximate normality of data using the Shapiro–Wilk test, descriptive statistics for anthropometric, biochemical, and respiratory muscle parameters are presented by arithmetic mean (standard deviation; SD), median (min-max), and by ratio (% and count). Between-groups analysis was performed using univariate analysis of covariance (ANCOVA) with age, level of physical activity, and BMI as covariates. Post hoc comparisons were corrected with the Bonferroni test. A Pearson chi-square test was used for gender and residential status in order to detect all between-group differences.

Linear regression analysis between VPT, DNS, and HbA1c on the one hand and measures of respiratory muscle strength and function (i.e., PI_max_, PE_max_, and PEF) on the other was performed with age as confounder.

## 3. Results

Subject characteristics are shown in Table 1. The healthy control group (C) was significantly younger (*F* = 3.487; *p* = 0.017), had lower BMI (*F* = 3.561; *p* = 0.015), was more physically active (*F* = 5.343; *p* = 0.002), and proportionally a minority was living in a residential setting (*p* = 0.002) compared to the other groups. There was no significant difference in gender distribution between the 3 groups (*p* = 0.587).

HbA1c levels were significantly higher in the diabetes groups compared to the control group (*F* = 24.894; *p* < 0.001), but showed no significant differences between the diabetes groups (D− vs. D+). Also, no significant between-group differences were found for duration of diabetes.

VPT-toe measures (left versus right) and VPT-ankle measures in the C, D−, and D+ are presented in Table 2.

The actual values of DNS reveal a score of zero on the scale of 0–4 in C in contrast with D+ (Table 3).

Linear regression analysis between VPT, DNS, and HbA1c on the one hand and measures of respiratory muscle strength and function (i.e., PI_max_, PE_max_, and PEF) on the other has been performed. Age was considered as a confounder since this is the single covariate strongly related to respiratory function, which is less so for the level of physical activity and BMI. The linear regression analysis on respiratory muscle strength (i.e., PI_max_ and PE_max_) shows that the VPT scores are the only significant explanatory values for the variances, respectively, in PI_max_ (8.2%) and PE_max_ (10.9%). Analyzing respiratory function (i.e., PEF), VPT (2.8%), Hb1Ac (5.5%), and age (17.5%) significantly explain 25.8% of the variance in PEF. The outcome data are documented in Table 4.

Table 5 reports on the assessment of respiratory muscle strength between the three groups, corrected for age, physical activity, and BMI. Significant differences were observed for PI_max_, PE_max_, and PEF. Post hoc analyses revealed significant lower values in D+ compared to C for PI_max_ (*p* = 0.005), PE_max_ (*p* = 0.001), and PEF (*p* < 0.001). When comparing D− with C, only PE_max_ (*p* = 0.039) and PEF (*p* = 0.026) were significantly lower.

Functional assessment data (i.e., HGS and SPPB) between the three groups are presented in Table 6, corrected for age, physical activity, and BMI.

HGS revealed no between-groups differences (*F* = 2.100; *p* = 0.128). Statistically significant differences were observed on both the SPPB total (*F* = 7.209; *p* = 0.001) as in its subdomains; CST (*F* = 4.533; *p* = 0.013), balance (*F* = 3.835; *p* = 0.025), and gait (*F* = 4.130; *p* = 0.019) with better performance in favor of C. For SPPB total and SPPB gait, post hoc analysis revealed significant higher values in C compared to D− (*p* = 0.008 and *p* = 0.043, respectively) and D+ (*p* = 0.002 and *p* = 0.031, respectively). CST and balance subdomains showed significant better scores for C compared to D+ (*p* = 0.010 and *p* = 0.028, respectively). Considering SPPB balance, only tandem stance showed significant higher results comparing C to D+ (*p* = 0.019).

## 4. Discussion

The present study was conducted to investigate respiratory muscle strength and function in T2DM and its relation to NP by comparing PI_max_, PE_max_, and PEF between T2DM patients with cdNP, T2DM patients without cdNP, and healthy controls.

The key findings of this study were lower measures of PI_max_, PE_max_, and PEF in the D− and D+ groups compared to C.

Looking more in detail to the results, all three respiratory muscle outcomes were significantly lower when comparing D+ to C; PE_max_ and PEF were significantly lower in D− and D+ compared to C. Herewith, it seems that the presence of NP as well as T2DM has an impact on respiratory muscle outcome. However, an accumulating effect of cdNP and T2DM could not be ascertained.

A posteriori power calculation on respiratory muscle strength and function (PI_max_, PE_max_, and PEF) resulted in a power of 0.826, 0.912, and 0.927, respectively.

To understand our NP-related results, the innervation of the respiratory muscles should be explored in depth. While breathing in, the inspiratory muscles contract by recruiting nonvolitional spinal nerves C3, C4, and C5 (the phrenic nerve) innervating the diaphragm, cranial nerve XI, spinal nerves C1 and C2 innervating the sternocleidomastoid and the scalene muscles, and T1 to T12 for the external intercostal muscles. The two expiratory muscle groups (the internal intercostals and abdominals) are usually not used during quiet breathing but are essential in performing expulsive efforts, including cough, vomiting, and defecation. Due to their character, these expiratory muscles are of utmost importance during forced expiration (such as in PE_max_ and PEF, during static and dynamic trunk control, and Valsalva maneuvers). The internal intercostal muscles are innervated by the spinal nerves T1 to T12 and the abdominals by spinal nerves T7 to L1.

The respiratory muscles are generally controlled by the respiratory centers of the autonomic nervous system in the pons and medulla oblongata and are depending on intact motor nerve supply, comparable to all skeletal muscles [45, 46].

Kabitz et al. used bilateral anterior magnetic phrenic nerve stimulation whereby the cortical motor command was bypassed in order to assess respiratory neuromuscular function related to diabetic poluneuropathy in patients with T2DM [4]. They provided the first data regarding cdNP and concluded that cdNP was associated with substantially impaired respiratory neuromuscular function in patients with T2DM, when stimulating the nonvolitional phrenic nerve. No alterations in respiratory function could be found when assessing volitional respiratory muscle strength. The volitional respiratory neuromuscular function testing was performed by using PI_max_, PE_max_, and maximal sniff pressures. PEF, however, was not assessed in this particular study. The exclusion criterion used by Kabitz et al. consisted in the diabetic group of known primary lung diseases, whereas in the control group healthy male subjects experiencing lung, cardiac, or metabolic diseases were excluded [4]. The different eligibility criteria between the research of Kabitz et al. and our own study could explain the different results. We opted for stricter selection criteria such as exclusion of patients with exercise-induced asthma and COPD GOLD stages 3 and 4. It is also of importance to mention the lower mean age of their controls (60.3 years ± 6.9) and the diabetic patients (63.6 years ± 7.5) compared to the present study, which could explain the differences in outcome of the volitional tests on respiratory neuromuscular function [47].

To understand the impact of T2DM as such, we have to focus on muscle mass, muscle fiber type distribution, and vascularization. Checking on the muscle fiber type distribution of the diaphragm in healthy humans, the mean relative occurrence of slow-twitch fibers (type I) is approximately 50%. The remaining proportion is equally divided into two different fast-twitch fibers (type IIa and IIx). Both the inspiratory and expiratory intercostal muscles have at least 10% more type I fibers than the diaphragm and most other skeletal muscles, whereas the expiratory internal intercostal muscles show an almost complete absence of type IIx fibers [46].

In healthy humans, all muscle fibers are surrounded by a certain number of capillaries, depending on their fiber type. In the diaphragm, type I fibers are surrounded by 4–6 capillaries per fiber, whereas slightly less [3–5] are found around type IIa and IIx fibers. However, the calculated values for the fiber area surrounded by each capillary are smaller in the diaphragm than in lower or upper limb muscles. In the expiratory intercostal muscles, more capillaries are found around both type I and type IIa fibers [5, 6] compared to the inspiratory intercostal muscles [4, 5, 46].

In the elderly in general and/or older T2DM patients, abnormalities in muscle morphology have been observed [48, 49]. Studies examining ageing and “accelerated” ageing in the older T2DM patients showed a reduced muscle mass and a decrease in muscle fiber size and number compared to younger controls [50–52]. Fiber size differences, particularly in the type II muscle fibers, seemed to be evident between healthy young men, healthy older men, and older age-matched T2DM patients, suggesting that type II fibers are more prone to muscle atrophy in the latter groups. When examining muscle capillary density (as a parameter of microvascular function), capillaries tended to be less prevalent in the elderly and/or older T2DM patients, implicating lower muscle capillary density. Dilation of these small capillaries could explain the observed shift in the distribution of vessel size with a relative loss of small vessels [52].

Measures of peripheral muscle strength (HGS) and functional performance (SPPB total with CST, balance, and gait as assessment tools) showed a similar profile as the respiratory muscle strength assessments; i.e., C scored better compared to D− and D+.

Analysis of the peripheral muscle strength showed no significant difference in HGS.

SPPB total, showed a significant difference (*p* = 0.001), mainly allocated to gait (usual gait speed over 2m44; *p* = 0.019) since both post hoc analyses showed significant differences of C versus D+ and D−. Walking is a complex motor skill, involving interactions between sensory and motor attributes, but is essentially supported by appropriate muscle strength and balance. Our findings regarding both strength impairment (CST, *p* = 0.013) and total balance changes (*p* = 0.025) with its subtest “tandem stance” (*p* = 0.022) endorse the earlier research results in T2DM and cdNP on gait [53]. The argument that gait performance could be influenced by T2DM alone (without NP) has to be taken into consideration based on previous findings [2, 9]. van Sloten et al. suggested that walking in subjects with T2DM was strongly associated with peripheral NP and decreased muscle strength. This associative result could not be established in our study when T2DM patients were compared to controls [9]. It could be hypothesized that T2DM as such has the same detrimental effects as the presence of cdNP in T2DM on a functional capacity (SPPB total and gait).

Based on our data, we can but conclude that both T2DM and NP significantly influence respiratory muscle strength and function. It was, however, not possible to distinct the initial cause (i.e., neuropathic respiratory impairments or diabetes-related pathology) of decreased respiratory muscle strength and function in our T2DM population.

The linear regression analysis on respiratory muscle strength (i.e., PI_max_ and PE_max_) suggests that the VPT scores are the only significant explanatory values (respectively, 8.2% and 10.9% of the variances in PI_max_ and PE_max_) rather than HbA1c, age, and DNS. These results indicate that VPT scores have a larger impact on respiratory muscle strength, supporting the hypothesis that respiratory muscle weakness is due to NP. Analyzing respiratory function (i.e., PEF), VPT (2.8%), Hb1Ac (5.5%), and age (17.5%) significantly explain 25.8% of the variance in PEF. Skloot provided evidence that the ageing process, in the absence of lung disease, alters the intrinsic structure of the lung (changes in collagen fiber network) as well as the supportive extrapulmonary structures (decreased chest wall compliance, reduced curvature of the diaphragm, and loss of respiratory muscle mass). These age-related changes in respiratory mechanics lead to a reduction in expiratory flow and lung volumes and affect lung function [47]. An explanation of the higher impact of HbA1c compared to the VPT scores can be found in the association between increased chronic glycemic exposure to the lung parenchyma and reduced pulmonary function in patients with T2DM [54].

The fact that all existing screening tools and questionnaires only rely on the measurements of appendicular muscles, and based on our results, it should be taken into consideration to integrate PI_max_, PE_max_, and PEF in the screening for respiratory muscle weakness as an indication for the presence of NP in diabetic patients [33]. These findings are supported by Lecube et al. [54] claiming that specific cost-effective screening programs for lung impairment, performed by health care providers, should be investigated in further research.

In the clinical practice of a general practitioner, measurements of lung function (forced vital capacity, forced expired volume after 1 second, Tiffeneau index, and PEF) are already regularly applied, mainly in order to detect COPD or other related respiratory disorders. Additional evaluations of respiratory muscle outcomes, which are easy to manage and have low cost impact (e.g., a portable Peak Flow Meter), could be of additional value and of importance in the screening for NP in the T2DM population.

Overall, impairments of respiratory muscle strength and function (PI_max_, PE_max_, and PEF) were slightly more pronounced compared to those of peripheral muscle strength. Since this study had a cross-sectional design, it was not possible to draw any conclusions concerning the timing of the impact on respiratory or peripheral muscles.

The participants were not questioned concerning smoking condition and alcohol consumption, although this information could have an impact on lung function in general, more specific PEF values, and on peripheral NP. However, due to a large group of respiratory disorders (asthma and COPD GOLD stages 3 and 4) were implemented as exclusion criterion, the impact of lung diseases on PI_max_, PE_max_, and PEF was significantly reduced. Regarding alcohol consumption, no data were collected which could affect peripheral NP as well. Besides, interviewing subjects about their smoking and drinking habits often lead to ambiguous answers out of exclusion fear.

The cross-sectional design limits drawing conclusions regarding the timing of the impact of causative variables on outcome parameters. Consequently, future research focus on both longitudinal research and the evolution of physical markers and symptoms such as—in this particular study—onset of diabetes, NP, and respiratory and peripheral muscle weakness.

It stands to reason that 82 out of 110 diabetic patients (74.5%) were allocated to the NP group (D+) known as one of the major comorbidities in T2DM patients [1, 2, 55]. Firstly, it is worth mentioning that according to Andersen et al. the prevalence of cdNP increases from 8% in newly diagnosed patients to >40% after 10 years of diabetes [3]. In the present study, the mean duration of diabetes was above 10 years from onset in both D− and D+ groups.

Secondly, the enrolled patients with T2DM showed higher mean age, higher BMI, and lower levels of physical activity compared to controls. Ageing is a well-known nonmodifiable factor for the development of diabetes and lower muscle strength [50]. BMI has a negative impact on muscle strength in a population with insulin resistance (prediabetic situation) and diabetes type 2, which will manifest itself as a decrease of absolute and relative peak torque [56, 57]. Concerning physical activity, low levels have a negative impact on the development of diabetes and on lower muscle strength [57]. In our ANCOVA we encountered this barrier by adding age, BMI, and physical activity to add them as covariates.

Finally, peripheral cdNP was diagnosed by VPT in combination with DNS with a deficient comprehensive neurological examination. Hence, we strongly recommend the use of the Michigan Neuropathy Screening Instrument in future research in order to allocate T2DM patients with/without NP more accurately to the respective groups [58–60]. In addition to these clinical assessments, MRI can be used in the detection of symptomatic NP, and nerve conduction investigations can be performed by means of electroneuromyography to evaluate sensory action potential amplitude and sensory and motor conduction velocity to confirm the presence and the severity of NP [61]. The main drawbacks to MRI and ENMG techniques are the high costs regarding the number of participants (initially 190) and the subject discomfort.

## 5. Conclusions

Based on a substantial population, this research, focusing on respiratory muscle strength, could conclude that this strength is negatively influenced in T2DM patients with and without peripheral neuropathy. A summation effect in patients with diabetes and neuropathy could not be ascertained. Screening for this muscle characteristic may add value to daily clinical practice of T2DM patients in assessment and follow-up.

## Figures and Tables

**Table 1:** Subject characteristics.

	C (*n* = 35)	D− (*n* = 28)	D+ (*n* = 82)
Age (yrs)	73 (6.8)	79 (9.9)	**79 (9.1)** ^**a**^
BMI (kg/m^2^)	28 (4.0)	**31 (6.2)** ^**a**^	29 (5.3)
HbA1c (%)	5.5 (0.40)	**6.7 (0.81)** ^**a**^	**6.7 (1.24)** ^**a**^
Diabetes duration (yrs)	/	10.5 (8.34)	10.3 (8.64)
Level of physical activity	8.2 (1.24–32.19)	6.4 (0.00–38.35)	**2.6 (0.00–36.41)** ^**ab**^
Male : female			
(%)	43 : 57	39 : 61	34 : 66
*(count)*	15 : 20	11 : 17	28 : 54
Community-dwelling : RCS			
(%)	71 : 29	**29** : **71** ^**a**^	**37** : **63** ^**a**^
*(count)*	25 : 10	**08** : **20** ^**a**^	**30** : **52** ^**a**^

Data were expressed as mean (SD), with exception for the level of physical activity as median (min-max), gender, and residential status as ratio (% and count); C: healthy controls; D−: T2DM without cdNP; D+: T2DM with cdNP; yrs: years; RCS: residential care setting; HbA1c: glycated hemoglobin; ^a^ *p* < 0.05 compared to C; ^b^ *p* < 0.05 compared to D−.

**Table 2:** VPT scores.

VPT: highest voltage of the left versus right toe			
	**C (** **n** = 34**)**	**D− (** **n** = 26**)**	**D+ (** **n** = 74**)**
Means (SD)	20.2 (5.54)	20.3 (6.04)	37.5 (12.26)
min-max	10–38	10–34	10–50

VPT: highest voltage of the left versus right ankle			
	**C (** **n** = 34**)**	**D− (** **n** = 26**)**	**D+ (** **n** = 70**)**
Means (SD)	23.6 (6.50)	24.0 (7.36)	40.6 (11.60)
min-max	12–35	10–45	7–50

Data were expressed as mean (SD) and minimum-maximum (min-max); VPT: Vibration Perception Threshold; C: healthy controls; D−: T2DM without cdNP; D+: T2DM with cdNP.

**Table 3:** DNS scores.

	C (*n* = 26)	D− (*n* = 22)	D+ (*n* = 70)
Means (SD)	0 (0)	0 (0)	1.4 (1.35)
Median (min-max)	0 (0-0)	0 (0-0)	1 (0–4)

Data were expressed as mean (SD) and median (minimum-maximum); DNS: Diabetic Neuropathy Symptom score; C: healthy controls; D−: T2DM without cdNP; D+: T2DM with cdNP.

**Table 4:** Linear regression analysis between VPT, DNS, HbA1c and age on one hand, and PI_max_, PE_max_ and PEF on the other.

	PI_max_	PE_max_	PEF
Explanatory variables	(i) VPT	(i) VPT	(i) VPT (ii) HbA1c (iii) Age
Adjusted R square	0.082	0.109	0.258
*p* values	*p* = 0.003	*p* = 0.001	*p* < 0.001

PI_max_: Maximum Inspiratory Pressure; PE_max_: Maximum Expiratory Pressure; PEF: Peak Expiratory Flow; VPT: Vibration Perception Threshold; HbA1c: glycated hemoglobin.

**Table 5:** Univariate analysis of covariance (ANCOVA, corrected for age, body mass index, and physical activity) on respiratory muscle strength and function.

	*F* value *p* value	C (*n* = 35)	D− (*n* = 28)	D+ (*n* = 82)
PI_max_ (cm H_2_O)	*F* = 5.289 *p* = 0.007	64.5 (28.83)	40.7 (25.22)	**36.6 (23.71)** ^**a**^
PE_max_ (cm H_2_O)	*F* = 6.785 *p* = 0.002	100.6 (29.58)	**69.5 (29.97)** ^**a**^	**65.2 (31.20)** ^**a**^
PEF (L/min)	*F* = 10.600 *p* = 0.001	471.2 (132.27)	**330.9 (152.07)** ^**a**^	**314.5 (221.26)** ^**a**^

Data were expressed as mean (SD); C: healthy controls; D−: T2DM without cdNP; D+: T2DM with cdNP; PI_max_: Maximum Inspiratory Pressure; PE_max_: Maximum Expiratory Pressure; PEF: Peak Expiratory Flow; ^a^ *p* < 0.05 compared to C.

**Table 6:** Univariate analysis of covariance (ANCOVA, corrected for age, body mass index, and physical activity) on peripheral muscle strength, balance, and gait.

	*F* value *p* value	C (*n* = 35)	D− (*n* = 28)	D+ (*n* = 82)
HGS (kg)	*F* = 2.100 *p* = 0.128	26.9 (12.36)	20.1 (10.15)	17.6 (9.50)
SPPB: total	*F* = 7.209 *p* = 0.001	11 (4–12)	**7 (1–12)** ^**a**^	**6 (1–12)** ^**a**^
(A) CST	*F* = 4.533 *p* = 0.013	3 (0–4)	1 (0–4)	**0 (0–4)** ^**a**^
(B) Balance total	*F* = 3.835 *p* = 0.025	4 (3–4)	3.5 (0–4)	**3 (0–4)** ^**a**^
Side-by-side	*F* = 0.508 *p* = 0.603	2 (2-2)	2 (0–2)	2 (0–2)
Semitandem	*F* = 1.334 *p* = 0.268	2 (2-2)	2 (0–2)	2 (0–2)
Tandem	*F* = 3.966 *p* = 0.022	2 (1-2)	1.5 (0–2)	**1 (0–2)** ^**a**^
(C) Gait	*F* = 4.130 *P* = 0.019	4 (1–4)	**2 (1–4)** ^**a**^	**3 (1–4)** ^**a**^

Data were expressed as median (min-max), with exception for HGS as mean (SD); C: healthy controls; D−: T2DM without cdNP; D+: T2DM with cdNP; HGS: Handgrip Strength; SPPB: Short Physical Performance Battery; CST: chair stand test; ^a^ *p* < 0.05 compared to C.

## Data Availability

The data used to support the findings of this study (BirgitVanEetvelde_20180723_revision.zsav) are included within the supplementary information file(s) (available here).

## Supplementary Materials

Supplementary Materials Below are template examples that authors may use to write a Data Availability statement. It will often be appropriate to combine templates and edit them as appropriate. (1) The data used to support the findings (BirgitVanEetvelde_20180723.sav) of this study are included within the supplementary information file(s). Read less.
