# Supplementary material for: The Influence of Clinically Diagnosed Neuropathy on Respiratory Muscle Strength in Type 2 Diabetes Mellitus
Source: J Diabetes Res. 2018 Nov 29;2018:8065938. doi: 10.1155/2018/8065938 (PMC6304822; doi:10.1155/2018/8065938)
Supplement: Supplementary Materials — Below are template examples that authors may use to write a Data Availability statement. It will often be appropriate to combine templates and edit them as appropriate. (1) The data used to support the findings (BirgitVanEetvelde_20180723.sav) of this study are included within the supplementary information file(s). Read less. [file 8065938.f1.pdf]

CODE	groep	NP95rec3	leeftijd	vetperc	BMI	HbA1c_per	HbA1ccat	HbA1c_mm
CTR001	2,00	3	73	49,20	31,2	6,1	#LEEG!	43
CTR002	2,00	3	81	48,80	27,3	6,3	#LEEG!	46
CTR005	2,00	3	78	49,90	31,4	5,7	#LEEG!	39
CTR006	2,00	3	92	49,20	29,6	5,7	#LEEG!	39
CTR023	2,00	3	69	44,80	41,7	6,0	#LEEG!	42
CTR024	2,00	3	76	42,40	29,8	5,2	#LEEG!	33
CTR029	2,00	3	80	22,10	22,4	6,2	#LEEG!	44
CTR036	2,00	3	86	50,70	33,7	5,7	#LEEG!	39
CTR043	2,00	3	84	53,90	28,3	5,0	#LEEG!	31
CTR047	2,00	3	72	26,60	25,6	4,8	#LEEG!	29
CTR049	2,00	3	73	31,90	25,0	5,5	#LEEG!	37
CTR051	2,00	3	70	28,90	25,3	5,1	#LEEG!	32
CTR052	2,00	3	72	33,50	25,0	5,4	#LEEG!	36
CTR054	2,00	3	67	26,70	23,6	5,3	#LEEG!	34
CTR055	2,00	3	75	43,40	29,0	5,6	#LEEG!	38
CTR057	2,00	3	71	30,30	25,7	5,4	#LEEG!	36
CTR058	2,00	3	67	32,70	30,3	5,8	#LEEG!	40
CTR059	2,00	3	71	26,50	24,2	5,2	#LEEG!	33
CTR060	2,00	3	69	39,60	27,1	5,4	#LEEG!	36
CTR061	2,00	3	68	20,70	23,7	5,0	#LEEG!	31
CTR062	2,00	3	72	29,50	26,5	5,2	#LEEG!	33
CTR063	2,00	3	73	30,00	23,5	5,4	#LEEG!	36
CTR064	2,00	3	61	20,90	26,3	5,1	#LEEG!	32
CTR066	2,00	3	66	25,10	32,9	5,7	#LEEG!	39
CTR068	2,00	3	69	41,50	27,5	6,5	#LEEG!	48
CTR070	2,00	3	71	40,70	23,4	5,4	#LEEG!	36
CTR072	2,00	3	79	32,80	32,1	5,8	#LEEG!	40
CTR073	2,00	3	78	33,10	26,8	5,3	#LEEG!	34
CTR074	2,00	3	83	50,20	30,7	5,2	#LEEG!	33
CTR078	2,00	3	67	31,80	30,7	6,0	#LEEG!	42
CTR085	2,00	3	66	45,20	26,8	5,4	#LEEG!	36
CTR089	2,00	3	63	38,60	22,1	5,0	#LEEG!	31
CTR094	2,00	3	67	32,80	23,9	4,9	#LEEG!	30
CTR095	2,00	3	73	36,70	27,1	6,2	#LEEG!	44
CTR097	2,00	3	75	41,90	27,2	5,5	#LEEG!	37
DIA001	1,00	1	76	43,90	40,8	8,3	1	68
DIA002	1,00	1	70	41,80	26,0	6,1	0	43
DIA003	1,00	1	75	32,70	25,5	6,4	0	47
DIA005	1,00	1	91	54,10	28,5	6,8	1	51
DIA006	1,00	1	93	58,00	29,8	5,6	0	38
DIA007	1,00	1	91	9999,00	20,4	5,7	0	39
DIA008	1,00	1	76	32,10	26,9	5,5	0	37
DIA009	1,00	1	66	40,30	28,9	7,9	1	63
DIA011	1,00	1	84	34,70	24,7	6,3	0	45
DIA012	1,00	1	85	56,10	28,9	5,4	0	36
DIA013	1,00	1	81	58,10	51,0	5,8	0	40
DIA014	1,00	1	82	51,10	29,6	5,8	0	40
DIA015	1,00	1	80	54,50	32,9	5,8	0	40
DIA016	1,00	1	83	47,30	29,1	5,3	0	34

DIA017	1,00	0	88	51,30	28,6	6,7	1	50
DIA018	1,00	1	79	33,10	31,9	6,5	1	48
DIA019	1,00	1	68	50,00	33,1	7,5	1	58
DIA021	1,00	0	74	56,10	41,9	6,5	1	48
DIA022	1,00	1	80	49,30	24,7	9999,0	9999	9999
DIA023	1,00	1	76	31,10	23,2	6,4	0	46
DIA024	1,00	1	61	47,20	32,6	5,6	0	38
DIA025	1,00	1	85	9999,00	26,7	7,0	1	53
DIA027	1,00	1	85	42,40	27,9	9999,0	9999	9999
DIA028	1,00	1	86	39,70	23,8	9999,0	9999	9999
DIA029	1,00	0	72	30,50	31,3	8,3	1	67
DIA030	1,00	1	79	43,90	26,8	6,6	1	49
DIA031	1,00	1	87	47,50	26,2	6,4	0	46
DIA032	1,00	1	86	48,40	31,0	5,9	0	41
DIA033	1,00	1	90	43,10	36,3	7,5	1	58
DIA034	1,00	1	93	54,70	19,8	6,4	0	46
DIA035	1,00	1	73	42,10	34,0	6,4	0	46
DIA036	1,00	0	79	32,00	31,1	6,5	1	48
DIA038	1,00	1	77	47,50	28,2	6,8	1	51
DIA039	1,00	1	89	49,50	33,7	6,1	0	43
DIA041	1,00	1	91	46,30	24,7	6,5	1	48
DIA042	1,00	1	87	47,60	31,6	8,2	1	66
DIA043	1,00	0	94	46,00	22,1	6,1	0	43
DIA044	1,00	1	87	44,70	26,3	8,6	1	70
DIA045	1,00	1	81	51,00	36,4	7,4	1	56
DIA047	1,00	1	83	45,00	25,3	9,9	1	85
DIA048	1,00	1	81	49,30	31,8	6,3	0	45
DIA049	1,00	1	80	57,60	37,7	8,5	1	69
DIA050	1,00	1	75	53,20	36,4	6,0	0	42
DIA051	1,00	0	93	56,40	29,0	5,8	0	40
DIA052	1,00	1	68	34,10	28,8	5,1	0	32
DIA053	1,00	1	71	33,90	35,9	9,4	1	79
DIA054	1,00	1	84	41,30	31,0	5,9	0	41
DIA055	1,00	0	74	56,00	40,5	5,9	0	41
DIA056	1,00	1	76	36,00	27,1	6,0	0	42
DIA057	1,00	1	80	35,90	22,6	6,2	0	44
DIA058	1,00	1	91	45,00	27,3	6,2	0	44
DIA059	1,00	1	87	31,90	28,3	9,1	1	76
DIA060	1,00	1	92	38,80	24,8	6,3	0	45
DIA061	1,00	1	83	42,20	31,4	6,8	1	51
DIA062	1,00	0	89	33,00	25,2	6,8	1	51
DIA063	1,00	1	78	24,90	26,0	6,7	1	50
DIA064	1,00	1	71	29,40	20,4	7,1	1	54
DIA065	1,00	1	75	35,30	28,0	8,6	1	70
DIA066	1,00	1	63	29,30	24,8	5,3	0	34
DIA067	1,00	1	86	48,70	38,4	12,3	1	111
DIA069	1,00	1	76	53,90	37,9	8,5	1	69
DIA071	1,00	1	85	45,70	27,8	5,7	0	39
DIA072	1,00	0	83	29,40	24,3	5,4	0	36
DIA074	1,00	0	86	55,90	38,2	6,6	1	49

DIA075	1,00	0	64	24,80	28,2	7,0	1	53
DIA076	1,00	1	68	27,20	24,5	7,1	1	54
DIA077	1,00	1	92	51,80	26,9	6,2	0	44
DIA078	1,00	1	81	41,10	31,0	7,7	1	61
DIA081	1,00	1	90	32,00	24,2	6,8	1	51
DIA082	1,00	1	89	9999,00	29,3	6,9	1	52
DIA084	1,00	0	88	33,70	25,4	6,5	1	48
DIA086	1,00	0	89	52,60	28,9	6,9	1	52
DIA087	1,00	0	93	41,60	30,2	6,7	1	50
DIA088	1,00	1	70	53,80	40,0	6,3	0	45
DIA089	1,00	1	71	46,20	31,7	5,3	0	34
DIA090	1,00	1	65	46,10	29,9	8,2	1	66
DIA092	1,00	0	69	27,00	26,8	8,1	1	65
DIA093	1,00	1	82	31,50	27,7	7,5	1	58
DIA094	1,00	1	67	45,50	32,8	5,6	0	38
DIA095	1,00	0	62	56,50	45,7	6,2	0	44
DIA096	1,00	1	79	27,20	23,3	6,1	0	43
DIA097	1,00	1	74	49,90	35,5	7,2	1	55
DIA099	1,00	1	69	36,70	33,8	6,8	1	51
DIA100	1,00	0	62	54,00	41,1	7,5	1	58
DIA101	1,00	1	73	43,20	31,1	7,0	1	53
DIA103	1,00	0	77	15,90	26,8	8,5	1	69
DIA104	1,00	1	92	38,50	26,3	6,7	1	50
DIA105	1,00	0	83	50,80	28,2	7,4	1	57
DIA106	1,00	1	69	30,00	23,2	5,8	0	40
DIA107	1,00	0	78	44,50	26,0	6,4	0	46
DIA108	1,00	0	73	28,90	30,3	6,1	0	43
DIA109	1,00	1	68	41,00	26,3	5,9	0	41
DIA110	1,00	1	63	52,80	32,8	6,1	0	43
DIA111	1,00	0	94	47,50	33,9	5,6	0	38
DIA112	1,00	1	77	33,80	26,8	5,6	0	38
DIA113	1,00	1	74	33,10	25,7	6,6	1	49
DIA114	1,00	1	63	48,40	34,1	9,3	1	78
DIA115	1,00	0	79	46,20	33,5	9999,0	9999	9999
DIA116	1,00	1	96	41,90	22,9	6,1	0	43
DIA117	1,00	1	77	46,00	28,6	5,3	0	34
DIA118	1,00	1	74	49,70	33,1	7,2	1	55
DIA119	1,00	0	69	58,20	43,0	5,6	0	38
DIA120	1,00	0	80	50,00	32,5	6,1	0	43
DIA121	1,00	0	75	51,80	27,8	7,4	1	57
DIA122	1,00	0	64	31,00	27,9	6,1	0	43
DIA123	1,00	1	64	20,00	21,8	5,6	0	38
DIA124	1,00	1	60	30,20	26,0	6,3	0	45
DIA125	1,00	1	75	49,90	33,9	6,4	0	46
DIA126	1,00	0	79	38,70	30,3	6,9	1	52
DIA127	1,00	1	61	22,10	26,5	7,1	1	54

duurdiabet	TOTAALBae	geslachtm1	verblijf0thu	HKKmax	@2m44	tandem	smal	semitander
9999,00	11,53	2	1	17	3,5	10,00	10,00	10,00
9999,00	7,30	2	1	20	3,5	10,00	10,00	10,00
9999,00	3,06	2	1	12	5,5	10,00	10,00	10,00
9999,00	3,21	2	1	6	6,5	7,10	10,00	10,00
9999,00	2,34	1	1	26	3,0	10,00	10,00	10,00
9999,00	7,84	2	1	12	2,0	5,00	10,00	10,00
9999,00	1,24	2	1	9999	4,0	6,00	10,00	10,00
9999,00	2,66	2	1	10	9999,0	9999,00	9999,00	9999,00
9999,00	9999,00	2	1	9999	4,4	10,00	10,00	10,00
9999,00	5,94	1	0	39	2,1	10,00	10,00	10,00
9999,00	7,88	1	0	22	1,8	10,00	10,00	10,00
9999,00	4,81	2	0	40	2,6	10,00	10,00	10,00
9999,00	11,84	1	1	19	3,0	10,00	10,00	10,00
9999,00	9,32	1	0	28	2,7	10,00	10,00	10,00
9999,00	6,68	2	0	16	2,1	10,00	10,00	10,00
9999,00	3,79	1	0	45	1,9	10,00	10,00	10,00
9999,00	14,35	1	0	54	1,8	10,00	10,00	10,00
9999,00	18,76	1	0	42	2,2	10,00	10,00	10,00
9999,00	9999,00	2	0	14	2,2	10,00	10,00	10,00
9999,00	9999,00	1	0	40	1,8	10,00	10,00	10,00
9999,00	9,47	1	0	42	1,4	10,00	10,00	10,00
9999,00	14,14	1	0	30	2,5	10,00	10,00	10,00
9999,00	9999,00	1	0	45	1,8	10,00	10,00	10,00
9999,00	9,51	1	0	47	2,0	10,00	10,00	10,00
9999,00	9999,00	2	0	18	2,2	10,00	10,00	10,00
9999,00	9,10	2	0	22	2,5	10,00	10,00	10,00
9999,00	15,24	1	0	26	2,3	10,00	10,00	10,00
9999,00	11,53	1	0	38	2,1	10,00	10,00	10,00
9999,00	9999,00	2	0	20	2,6	10,00	10,00	10,00
9999,00	5,84	2	0	20	1,8	10,00	10,00	10,00
9999,00	9999,00	2	0	24	1,8	10,00	10,00	10,00
9999,00	9999,00	2	0	30	1,9	10,00	10,00	10,00
9999,00	32,19	2	0	20	2,2	10,00	10,00	10,00
9999,00	8,55	2	0	26	1,8	10,00	10,00	10,00
9999,00	9999,00	2	0	19	2,3	10,00	10,00	10,00
10,00	1,36	1	0	22	3,5	0,00	10,00	10,00
16,00	7,78	2	0	21	3,0	10,00	10,00	10,00
4,00	9999,00	1	0	22	3,7	0,00	10,00	10,00
9999,00	3,95	2	1	14	4,1	10,00	10,00	10,00
5,00	1,14	2	1	11	10,8	0,00	10,00	10,00
15,00	5,62	2	1	8	6,2	6,84	10,00	10,00
35,00	3,16	1	0	39	3,8	0,00	1,00	0,00
9999,00	9999,00	2	0	14	2,4	10,00	10,00	10,00
0,17	0,91	1	1	24	3,3	10,00	10,00	10,00
9999,00	9999,00	2	1	10	3,4	0,00	10,00	10,00
20,00	3,19	2	1	16	3,7	9,50	10,00	10,00
6,00	1,84	2	1	5	5,1	10,00	10,00	10,00
5,00	4,18	2	1	15	3,0	10,00	10,00	10,00
9999,00	9999,00	2	1	7	5,0	9999,00	10,00	9999,00

25,00	9999,00	2	1	16	4,7	0,00	3,00	0,00
5,00	4,90	1	0	23	3,0	2,32	10,00	10,00
20,00	9,84	2	0	22	3,1	10,00	10,00	10,00
9999,00	9999,00	2	1	16	4,1	10,00	10,00	10,00
8,00	1,14	2	1	12	9999,0	0,00	0,00	0,00
2,00	1,14	1	1	30	2,2	10,00	10,00	10,00
7,00	6,32	2	1	9999	4,8	0,00	10,00	0,00
3,00	3,19	2	1	10	9,2	0,00	0,00	0,00
10,00	1,14	2	1	9999	8,5	6,53	10,00	10,00
9999,00	9999,00	2	1	8	13,3	0,00	0,00	0,00
4,00	6,36	1	1	24	4,2	10,00	10,00	10,00
10,00	4,16	2	1	10	5,4	10,00	10,00	10,00
9999,00	6,99	2	1	12	4,1	0,00	0,00	0,00
12,00	1,65	2	1	8	6,3	0,00	10,00	10,00
8,00	0,00	2	1	8	8,1	0,00	10,00	10,00
9999,00	1,14	2	1	9999	12,1	6,06	10,00	10,00
6,00	1,77	1	1	28	3,4	9999,00	10,00	10,00
35,00	9,61	1	1	25	3,4	0,00	10,00	10,00
5,00	4,15	2	1	18	3,5	3,50	10,00	10,00
10,00	1,61	2	1	16	8,5	0,00	10,00	0,00
5,00	0,00	2	1	9999	3,6	9999,00	10,00	10,00
5,00	4,45	2	1	15	3,7	9999,00	10,00	10,00
6,00	1,89	2	1	5	8,4	9999,00	10,00	10,00
22,00	2,00	2	1	11	4,6	5,00	10,00	10,00
20,00	1,14	2	1	18	8,4	0,00	10,00	10,00
3,00	1,78	2	1	6	6,7	0,00	10,00	10,00
6,00	1,77	2	1	10	12,2	9999,00	10,00	10,00
4,00	0,00	2	1	9999	9,9	1,00	10,00	10,00
6,00	3,72	2	1	18	5,1	10,00	10,00	10,00
9999,00	9999,00	2	1	9999	6,7	0,00	10,00	10,00
10,00	0,34	1	1	10	3,4	0,00	10,00	0,00
9999,00	2,47	1	1	20	4,8	0,00	10,00	10,00
9999,00	9999,00	1	1	8	6,7	1,69	10,00	10,00
5,00	1,92	2	1	9999	6,1	0,00	0,00	0,00
4,00	0,00	1	1	18	5,1	1,50	10,00	10,00
4,00	1,21	2	1	8	4,4	10,00	10,00	10,00
17,00	1,85	2	1	9999	3,7	2,00	10,00	10,00
9999,00	9999,00	1	1	18	3,5	0,00	0,00	0,00
5,00	1,14	1	1	22	3,8	10,00	10,00	10,00
6,00	2,27	2	1	12	7,1	8,80	10,00	10,00
8,00	7,18	1	1	25	2,7	9999,00	10,00	10,00
3,00	3,41	1	0	19	4,4	3,16	10,00	10,00
22,00	10,17	1	0	36	3,2	10,00	10,00	10,00
5,00	11,87	1	0	35	3,7	3,31	10,00	10,00
9999,00	9999,00	1	0	28	2,9	9,50	10,00	10,00
35,00	4,91	2	1	6	7,5	9999,00	10,00	10,00
9,00	2,78	2	1	5	4,0	0,00	10,00	0,00
9999,00	9999,00	2	1	6	6,3	9999,00	10,00	10,00
9999,00	9999,00	1	1	23	4,1	3,00	10,00	10,00
2,00	16,63	2	1	7	2,8	1,00	10,00	10,00

12,00	10,59	1	0	38	1,9	10,00	10,00	10,00
17,00	28,00	1	0	18	2,4	10,00	10,00	10,00
10,00	5,43	2	1	11	4,8	10,00	10,00	10,00
3,00	1,42	1	1	15	9999,0	0,00	0,00	0,00
2,00	3,45	1	1	16	4,0	8,00	10,00	10,00
3,00	5,71	2	1	8	5,5	9999,00	10,00	10,00
4,00	3,87	1	1	17	4,5	0,00	10,00	10,00
9999,00	9999,00	2	1	8	7,5	10,00	10,00	10,00
5,00	3,46	2	1	5	6,0	0,00	8,00	6,00
18,00	3,55	2	0	20	3,0	3,72	10,00	10,00
10,00	1,40	2	0	20	2,1	10,00	10,00	10,00
9999,00	9999,00	2	0	18	2,3	10,00	10,00	10,00
17,00	20,18	1	0	34	2,4	10,00	10,00	10,00
35,00	5,76	1	0	31	3,1	10,00	10,00	10,00
9,00	6,11	2	0	24	3,0	10,00	10,00	10,00
8,00	10,38	2	0	18	2,6	10,00	10,00	10,00
8,00	16,08	1	0	24	2,8	4,84	10,00	10,00
20,00	1,70	2	0	10	3,7	10,00	10,00	10,00
9999,00	6,39	1	0	11	4,5	10,00	10,00	10,00
9999,00	5,83	2	0	20	3,8	10,00	10,00	10,00
25,00	1,70	2	0	18	3,9	10,00	10,00	10,00
9999,00	9999,00	1	0	23	3,0	10,00	10,00	10,00
9999,00	9999,00	1	1	14	2,8	7,00	10,00	10,00
9999,00	9999,00	2	1	9	6,3	0,50	10,00	10,00
16,00	12,50	2	0	13	2,3	10,00	10,00	10,00
12,00	7,25	2	0	18	2,8	10,00	10,00	10,00
8,00	12,36	1	0	42	2,1	10,00	10,00	10,00
8,00	7,24	2	0	24	2,8	4,30	10,00	10,00
9999,00	0,80	2	1	14	2,8	2,47	10,00	3,72
9999,00	9,45	2	1	25	9999,0	10,00	10,00	10,00
7,00	4,95	1	0	30	3,3	10,00	10,00	10,00
35,00	0,60	2	0	14	6,4	10,00	10,00	10,00
0,50	0,90	2	0	20	3,1	3,41	10,00	10,00
14,00	0,34	2	1	18	4,4	5,62	10,00	10,00
3,00	0,54	2	1	16	5,9	0,00	10,00	1,00
4,00	1,19	2	1	12	9999,0	0,00	10,00	10,00
3,00	2,26	2	1	13	6,2	0,00	10,00	10,00
5,00	38,35	2	1	26	4,1	10,00	10,00	10,00
7,00	0,00	2	1	12	9,8	8,50	10,00	10,00
17,00	4,80	2	1	8	7,9	1,00	10,00	10,00
1,00	2,87	1	1	25	4,3	2,00	10,00	10,00
8,00	6,32	1	0	36	2,2	10,00	10,00	10,00
10,00	5,68	1	0	48	1,6	10,00	10,00	10,00
5,00	2,58	2	0	26	2,3	10,00	10,00	10,00
15,00	4,00	1	0	36	2,0	10,00	10,00	10,00
9,00	36,41	1	0	50	2,1	10,00	10,00	10,00

TCRaantal	TCRtime	MPInspma	MPExpmax	piekflowma	lengtecm	LGkg	waterperc	impedantie
0,00	9999,00	31,67	54,95	460,00	166,0	86,1	39,80	621,00
5,00	12,50	58,52	85,16	400,00	150,0	61,4	46,50	619,00
0,00	9999,00	41,76	80,40	480,00	158,0	78,4	42,00	584,00
0,00	9999,00	20,02	33,30	370,00	150,0	66,5	47,40	544,00
0,00	9999,00	55,57	127,55	450,00	161,0	108,2	40,80	508,00
5,00	24,60	31,92	72,57	250,00	162,0	78,1	46,70	612,00
0,00	9999,00	18,64	50,25	250,00	145,0	47,0	77,40	347,00
9999,00	9999,00	16,48	94,28	260,00	150,0	75,8	44,70	487,00
0,00	9999,00	9999,00	9999,00	190,00	148,0	61,9	43,60	705,00
5,00	2,10	96,51	133,30	550,00	172,0	75,6	55,20	521,00
5,00	15,05	37,22	70,34	550,00	176,0	77,4	51,20	611,00
5,00	11,10	55,52	118,59	690,00	173,0	75,8	54,40	548,00
5,00	17,80	9999,00	9999,00	9999,00	159,0	63,3	54,50	588,00
5,00	13,50	78,97	105,69	490,00	167,0	65,9	58,90	528,00
5,00	11,30	82,66	75,60	410,00	155,0	69,6	46,40	552,00
5,00	10,50	113,35	171,00	630,00	171,0	75,2	50,90	604,00
5,00	11,50	129,51	124,89	720,00	169,0	86,6	55,40	416,00
5,00	12,60	39,77	96,27	650,00	170,0	69,9	58,70	514,00
5,00	16,40	68,42	123,40	500,00	159,0	68,5	49,60	523,00
5,00	8,70	69,50	111,34	520,00	169,0	67,6	64,90	444,00
5,00	10,00	88,76	114,04	660,00	169,0	75,6	52,00	563,00
5,00	10,50	120,76	119,65	670,00	171,0	68,6	55,40	586,00
5,00	11,50	9999,00	9999,00	510,00	166,0	72,6	62,80	408,00
5,00	10,90	67,96	96,52	550,00	170,0	95,1	57,60	340,00
5,00	19,00	49,77	63,97	390,00	165,0	75,0	46,10	564,00
5,00	10,70	57,64	97,01	430,00	158,0	58,3	50,60	624,00
5,00	14,00	49,71	97,03	380,00	174,0	97,2	52,40	420,00
5,00	8,80	61,32	99,40	600,00	173,0	80,1	51,10	569,00
5,00	19,80	37,73	90,39	350,00	159,0	77,7	42,10	597,00
5,00	13,20	91,24	122,44	480,00	164,0	82,6	55,10	361,00
5,00	9,60	72,09	89,47	500,00	156,0	65,2	45,10	645,00
5,00	12,60	73,51	154,97	450,00	162,0	58,0	49,50	701,00
5,00	10,00	89,92	130,91	480,00	166,0	65,8	53,50	527,00
5,00	9,20	72,44	110,37	340,00	166,0	74,7	49,30	511,00
5,00	11,50	84,80	103,31	410,00	159,0	68,8	48,50	550,00
5,00	29,00	9999,00	9999,00	9999,00	176,0	126,5	42,70	447,00
5,00	18,57	58,47	83,64	380,00	165,0	70,8	45,90	623,00
5,00	17,91	9999,00	9999,00	9999,00	160,0	65,2	55,50	548,00
0,00	9999,00	27,19	73,01	320,00	159,0	72,1	40,20	739,00
0,00	9999,00	26,50	55,97	150,00	144,0	61,7	42,80	684,00
0,00	9999,00	41,74	49,53	200,00	156,0	49,7	9999,00	9999,00
0,00	9999,00	75,49	117,25	480,00	174,0	81,5	51,80	544,00
5,00	14,78	87,18	101,79	370,00	156,0	70,4	49,00	495,00
0,00	9999,00	36,85	98,02	400,00	166,0	68,0	54,10	585,00
0,00	9999,00	16,84	26,56	100,00	140,0	56,7	45,30	658,00
0,00	9999,00	39,30	64,38	240,00	154,0	121,0	36,00	404,00
0,00	9999,00	48,85	97,21	270,00	156,0	72,0	42,10	643,00
5,00	31,00	16,42	81,27	350,00	151,0	75,1	39,70	641,00
9999,00	9999,00	20,54	46,42	320,00	158,0	72,7	45,10	565,00

0,00	9999,00	20,54	46,42	320,00	162,0	75,0	41,30	678,00
5,00	31,50	87,57	116,74	340,00	181,0	104,5	51,20	430,00
5,00	14,90	9999,00	9999,00	400,00	159,0	83,8	40,10	587,00
5,00	31,20	38,66	49,86	180,00	154,0	99,4	37,10	499,00
0,00	9999,00	28,87	41,21	90,00	156,0	60,0	46,00	674,00
5,00	17,90	9999,00	9999,00	9999,00	178,0	73,6	53,80	567,00
0,00	9999,00	9999,00	9999,00	9999,00	156,0	79,4	41,90	618,00
0,00	9999,00	9,66	29,01	9999,00	152,0	61,7	9999,00	9999,00
0,00	9999,00	22,87	38,26	170,00	157,0	68,8	50,60	490,00
0,00	9999,00	9999,00	9999,00	185,00	153,0	55,7	57,10	508,00
5,00	25,50	19,76	38,63	230,00	172,0	92,5	53,20	439,00
0,00	9999,00	14,18	73,35	100,00	154,0	63,6	49,50	550,00
0,00	9999,00	44,98	57,46	210,00	161,0	67,8	45,40	647,00
0,00	9999,00	16,51	78,35	280,00	154,0	73,5	45,70	515,00
0,00	9999,00	41,83	48,18	220,00	154,0	86,0	51,00	342,00
0,00	9999,00	23,28	36,44	200,00	143,0	40,4	58,20	711,00
0,00	9999,00	28,74	58,42	280,00	175,0	104,1	41,40	613,00
0,00	9999,00	42,33	110,31	490,00	180,0	100,7	52,00	435,00
0,00	9999,00	32,99	78,05	360,00	161,0	73,2	43,20	635,00
0,00	9999,00	15,07	49,57	130,00	156,0	82,0	44,30	486,00
0,00	9999,00	7,19	33,15	120,00	163,0	65,7	47,00	648,00
0,00	9999,00	44,96	35,21	170,00	158,0	78,8	44,70	519,00
0,00	9999,00	8,12	47,90	160,00	156,0	53,7	52,80	636,00
5,00	15,00	31,97	92,50	200,00	142,0	53,0	53,80	599,00
0,00	9999,00	32,11	68,41	220,00	150,0	82,0	41,00	511,00
0,00	9999,00	12,65	49,70	200,00	158,0	63,1	47,10	678,00
0,00	9999,00	22,29	35,34	180,00	159,0	80,4	42,00	587,00
0,00	9999,00	11,62	31,98	70,00	157,0	93,0	35,30	655,00
0,00	9999,00	57,24	55,12	290,00	154,0	86,4	39,20	563,00
0,00	9999,00	26,61	44,86	80,00	152,0	67,1	40,50	753,00
0,00	9999,00	11,99	35,38	250,00	165,0	78,5	49,30	572,00
0,00	9999,00	21,19	52,23	350,00	160,0	91,8	49,90	428,00
0,00	9999,00	25,09	75,42	160,00	148,0	68,0	49,90	557,00
0,00	9999,00	11,19	30,34	150,00	147,0	87,6	38,30	536,00
0,00	9999,00	76,96	91,58	470,00	174,0	82,0	47,00	650,00
0,00	9999,00	25,95	65,45	90,00	153,0	52,9	58,70	531,00
0,00	9999,00	55,72	103,50	300,00	159,0	69,0	47,00	567,00
0,00	9999,00	15,28	79,55	270,00	158,0	70,6	58,60	444,00
5,00	27,00	83,00	84,17	410,00	158,0	61,9	54,30	624,00
5,00	12,50	43,87	98,47	300,00	154,0	74,4	50,10	437,00
0,00	9999,00	9999,00	9999,00	9999,00	173,0	75,5	54,00	547,00
5,00	18,20	86,90	110,94	470,00	164,0	69,8	63,20	415,00
5,00	8,90	73,03	99,37	500,00	168,0	57,5	61,00	607,00
5,00	9,00	53,00	103,89	370,00	169,0	79,9	49,10	584,00
5,00	18,95	54,24	101,25	430,00	162,0	65,2	55,50	569,00
0,00	9999,00	23,29	63,13	250,00	164,0	103,4	43,30	401,00
0,00	9999,00	17,53	34,14	120,00	151,0	86,5	39,90	510,00
0,00	9999,00	15,26	42,03	200,00	157,0	68,5	47,40	556,00
0,00	9999,00	48,89	97,51	470,00	175,0	74,5	56,90	520,00
0,00	9999,00	30,48	66,31	270,00	165,0	104,1	36,20	579,00

5,00	12,05	112,13	131,30	440,00	170,0	81,5	56,70	438,00
5,00	11,95	69,08	159,45	500,00	166,0	67,5	58,40	517,00
5,00	46,00	55,58	56,61	250,00	156,0	65,5	43,90	685,00
0,00	9999,00	8,76	26,59	9999,00	155,0	74,5	47,20	589,00
5,00	17,00	68,03	86,22	370,00	170,0	70,0	57,40	532,00
0,00	9999,00	9999,00	9999,00	9999,00	149,0	65,0	9999,00	9999,00
0,00	9999,00	47,91	58,60	600,00	169,0	72,6	54,70	542,00
0,00	9999,00	16,89	69,92	300,00	150,0	65,0	44,60	623,00
0,00	9999,00	39,68	63,95	130,00	155,0	72,6	53,20	405,00
5,00	11,50	41,88	94,17	230,00	159,0	101,1	37,60	503,00
5,00	11,80	41,08	118,88	400,00	157,0	78,1	44,30	522,00
5,00	15,00	37,98	77,89	360,00	160,0	76,6	42,60	602,00
5,00	16,00	72,05	118,84	580,00	172,0	79,3	56,20	471,00
5,00	14,50	59,25	91,18	270,00	172,0	81,8	54,20	485,00
5,00	13,00	57,54	64,83	330,00	167,0	91,4	42,30	512,00
5,00	17,20	86,35	105,95	450,00	159,0	115,6	35,60	463,00
5,00	16,00	97,84	81,08	550,00	177,0	72,9	58,20	533,00
5,00	32,00	23,70	45,58	230,00	157,0	87,4	41,60	498,00
0,00	9999,00	23,67	82,56	350,00	171,0	98,7	46,70	497,00
5,00	14,00	71,35	101,71	490,00	158,0	102,5	36,90	511,00
5,00	13,00	27,85	110,05	380,00	168,0	87,9	44,80	494,00
5,00	14,00	55,42	98,75	390,00	177,0	84,1	69,30	328,00
0,00	9999,00	26,78	90,25	420,00	160,0	67,2	53,10	573,00
0,00	9999,00	31,74	53,71	350,00	159,0	71,4	41,90	688,00
5,00	15,10	9999,00	9999,00	320,00	160,0	59,3	57,70	497,00
5,00	41,45	38,00	63,28	360,00	153,0	60,9	49,00	579,00
5,00	14,56	35,13	93,14	560,00	171,0	88,5	55,70	410,00
5,00	35,86	45,18	45,78	330,00	164,0	70,8	47,20	575,00
5,00	10,53	28,82	36,45	280,00	159,0	83,0	36,90	711,00
5,00	11,88	65,70	97,41	460,00	158,0	84,6	41,30	538,00
5,00	7,00	81,10	85,27	490,00	175,0	82,0	51,80	469,00
5,00	48,00	14,84	27,51	380,00	160,0	65,8	56,40	448,00
5,00	19,00	53,28	64,79	250,00	155,0	81,9	41,60	534,00
5,00	11,25	32,66	54,56	180,00	155,0	80,6	46,40	445,00
0,00	9999,00	14,19	12,03	110,00	163,0	60,8	53,00	579,00
9999,00	9999,00	7,78	18,96	150,00	157,0	70,6	45,90	566,00
5,00	24,00	23,06	33,31	160,00	151,0	75,5	43,40	526,00
5,00	32,31	13,04	48,82	320,00	162,0	112,8	33,70	564,00
2,00	27,75	46,42	48,72	224,00	153,0	76,0	42,30	578,00
5,00	40,06	40,05	47,82	9999,00	148,0	60,8	43,60	709,00
5,00	12,25	9999,00	9999,00	150,00	163,0	74,0	52,30	529,00
5,00	10,00	11,07	12,31	575,00	178,0	69,0	64,70	474,00
5,00	10,18	14,89	38,12	560,00	174,0	78,7	50,40	599,00
5,00	12,25	5,67	4,87	140,00	161,0	88,0	41,00	536,00
5,00	9,78	6,19	17,38	270,00	162,0	79,6	48,00	565,00
5,00	13,36	6,85	18,98	600,00	177,0	82,9	59,00	433,00

Baeckehuis	Baeckespoı	BaeckeVrij	laagsteVPT	laagsteVPT	hoogsteVP	laagsteVPT	laagsteVPT	hoogsteVP
1,70	2,87	6,96	16,0	18,0	18,0	17,0	24,0	24,0
1,80	4,40	1,09	18,0	15,0	18,0	10,0	17,0	17,0
0,60	1,23	1,23	19,0	24,0	24,0	30,0	30,0	30,0
0,70	0,19	2,32	9999,0	9999,0	9999,0	9999,0	9999,0	9999,0
1,20	0,00	1,14	26,0	22,0	26,0	35,0	32,0	35,0
0,30	0,00	7,54	18,0	18,0	18,0	22,0	20,0	22,0
0,10	0,00	1,14	20,0	23,0	23,0	19,0	18,0	19,0
0,00	0,00	2,66	38,0	31,0	38,0	32,0	24,0	32,0
9999,00	9999,00	9999,00	18,0	16,0	18,0	30,0	18,0	30,0
2,10	0,00	3,84	17,0	18,0	18,0	22,0	20,0	22,0
2,30	5,58	0,00	21,0	26,0	26,0	35,0	31,0	35,0
2,20	2,61	0,00	19,0	20,0	20,0	29,0	17,0	29,0
1,10	0,00	10,74	21,0	20,0	21,0	20,0	19,0	20,0
1,50	7,82	0,00	18,0	20,0	20,0	18,0	17,0	18,0
3,00	3,68	0,00	10,0	10,0	10,0	11,0	15,0	15,0
2,10	0,00	1,69	23,0	14,0	23,0	25,0	16,0	25,0
1,70	2,87	9,78	20,0	20,0	20,0	20,0	25,0	25,0
1,80	11,30	5,66	20,0	20,0	20,0	12,0	15,0	15,0
9999,00	9999,00	9999,00	19,0	20,0	20,0	19,0	20,0	20,0
9999,00	9999,00	9999,00	10,0	15,0	15,0	12,0	12,0	12,0
1,40	1,98	6,09	17,0	13,0	17,0	30,0	20,0	30,0
1,40	1,23	11,51	13,0	25,0	25,0	14,0	20,0	20,0
9999,00	9999,00	9999,00	11,0	15,0	15,0	14,0	13,0	14,0
2,30	0,00	7,21	10,0	19,0	19,0	30,0	29,0	30,0
9999,00	9999,00	9999,00	14,0	14,0	14,0	29,0	30,0	30,0
1,90	4,09	3,12	20,0	15,0	20,0	23,0	15,0	23,0
2,20	13,04	0,00	30,0	30,0	30,0	30,0	32,0	32,0
2,20	1,23	8,10	15,0	20,0	20,0	15,0	19,0	19,0
9999,00	9999,00	9999,00	20,0	32,0	32,0	34,0	34,0	34,0
2,00	0,00	3,84	14,0	13,0	14,0	20,0	21,0	21,0
9999,00	9999,00	9999,00	17,0	15,0	17,0	19,0	15,0	19,0
9999,00	9999,00	9999,00	20,0	21,0	21,0	18,0	19,0	19,0
2,70	9,56	19,93	17,0	18,0	18,0	20,0	19,0	20,0
1,90	4,50	2,16	9,0	15,0	15,0	29,0	28,0	29,0
9999,00	9999,00	9999,00	14,0	15,0	15,0	19,0	18,0	19,0
0,00	0,00	1,36	26,0	40,0	40,0	31,0	28,0	31,0
1,90	1,88	0,00	16,0	24,0	24,0	24,0	36,0	36,0
0,65	9999,00	9999,00	20,0	15,0	20,0	19,0	20,0	20,0
0,40	1,23	2,32	48,0	50,0	50,0	45,0	50,0	50,0
0,00	0,00	1,14	35,0	35,0	35,0	38,0	38,0	38,0
0,00	2,61	3,01	40,0	50,0	50,0	50,0	50,0	50,0
0,40	0,00	2,76	9999,0	9999,0	9999,0	9999,0	9999,0	9999,0
9999,00	9999,00	9999,00	50,0	40,0	50,0	43,0	28,0	43,0
0,50	0,00	0,41	38,0	25,0	38,0	28,0	35,0	35,0
9999,00	9999,00	9999,00	50,0	50,0	50,0	22,0	38,0	38,0
0,40	0,97	1,82	50,0	50,0	50,0	50,0	36,0	50,0
0,00	0,87	0,97	30,0	9999,0	30,0	20,0	50,0	50,0
0,90	0,00	3,28	40,0	20,0	40,0	40,0	40,0	40,0
9999,00	9999,00	9999,00	32,0	44,0	44,0	50,0	48,0	50,0

9999,00	9999,00	9999,00	20,0	19,0	20,0	21,0	25,0	25,0
1,40	2,69	0,81	30,0	50,0	50,0	50,0	9999,0	50,0
0,00	4,35	5,49	25,0	20,0	25,0	40,0	19,0	40,0
0,50	0,00	9999,00	21,0	28,0	28,0	30,0	30,0	30,0
0,00	0,00	1,14	6,0	10,0	10,0	7,0	6,0	7,0
0,00	0,00	1,14	42,0	40,0	42,0	9999,0	9999,0	9999,0
0,00	0,00	6,32	16,0	13,0	16,0	18,0	14,0	18,0
0,00	0,00	3,19	36,0	30,0	36,0	50,0	25,0	50,0
0,00	0,00	1,14	18,0	19,0	19,0	26,0	22,0	26,0
9999,00	9999,00	9999,00	40,0	24,0	40,0	50,0	48,0	50,0
0,40	0,00	5,96	14,0	19,0	19,0	20,0	14,0	20,0
0,40	0,00	3,76	24,0	46,0	46,0	49,0	46,0	49,0
0,80	6,09	0,10	50,0	38,0	50,0	50,0	50,0	50,0
0,00	0,00	1,65	22,0	28,0	28,0	28,0	50,0	50,0
0,00	0,00	0,00	30,0	20,0	30,0	18,0	20,0	20,0
0,00	0,00	1,14	20,0	50,0	50,0	50,0	50,0	50,0
0,00	0,00	1,77	9999,0	9999,0	9999,0	9999,0	9999,0	9999,0
0,10	9,05	0,46	9999,0	9999,0	9999,0	9999,0	9999,0	9999,0
0,40	2,61	1,14	9999,0	9999,0	9999,0	9999,0	9999,0	9999,0
0,00	0,00	1,61	9999,0	9999,0	9999,0	9999,0	9999,0	9999,0
0,00	0,00	0,00	9999,0	9999,0	9999,0	9999,0	9999,0	9999,0
0,00	0,00	4,45	9999,0	9999,0	9999,0	9999,0	9999,0	9999,0
0,00	0,00	1,89	9999,0	9999,0	9999,0	9999,0	9999,0	9999,0
0,00	0,00	2,00	50,0	50,0	50,0	50,0	9999,0	50,0
0,00	0,00	1,14	30,0	40,0	40,0	40,0	50,0	50,0
0,00	0,00	1,78	20,0	20,0	20,0	20,0	30,0	30,0
0,00	0,00	1,77	31,0	30,0	31,0	50,0	9999,0	50,0
0,00	0,00	0,00	9999,0	9999,0	9999,0	9999,0	9999,0	9999,0
0,60	0,00	3,12	37,0	20,0	37,0	50,0	36,0	50,0
9999,00	9999,00	9999,00	16,0	30,0	30,0	38,0	32,0	38,0
0,00	0,00	0,34	50,0	50,0	50,0	50,0	9999,0	50,0
0,70	0,00	1,77	40,0	20,0	40,0	9999,0	50,0	50,0
9999,00	9999,00	9999,00	30,0	50,0	50,0	50,0	20,0	50,0
0,00	0,00	1,92	20,0	21,0	21,0	20,0	22,0	22,0
0,00	0,00	0,00	10,0	15,0	15,0	39,0	20,0	39,0
0,00	0,00	1,21	20,0	25,0	25,0	27,0	19,0	27,0
0,30	0,00	1,55	30,0	29,0	30,0	21,0	28,0	28,0
9999,00	9999,00	9999,00	50,0	9999,0	50,0	9999,0	9999,0	9999,0
0,00	0,00	1,14	40,0	45,0	45,0	41,0	50,0	50,0
0,30	0,00	1,97	50,0	50,0	50,0	50,0	50,0	50,0
1,00	0,00	6,18	18,0	20,0	20,0	20,0	20,0	20,0
0,80	0,00	2,61	50,0	50,0	50,0	9999,0	50,0	50,0
1,20	0,87	8,10	45,0	50,0	50,0	50,0	40,0	50,0
0,30	0,00	11,57	20,0	50,0	50,0	25,0	40,0	40,0
9999,00	9999,00	9999,00	50,0	29,0	50,0	50,0	32,0	50,0
0,10	4,81	0,00	50,0	9999,0	50,0	50,0	50,0	50,0
1,00	0,00	1,78	30,0	22,0	30,0	30,0	42,0	42,0
9999,00	9999,00	9999,00	50,0	50,0	50,0	9999,0	9999,0	9999,0
9999,00	9999,00	9999,00	16,0	15,0	16,0	25,0	30,0	30,0
0,10	16,53	0,01	24,0	24,0	24,0	30,0	29,0	30,0

1,90	8,69	0,00	21,0	19,0	21,0	24,0	19,0	24,0
2,00	19,13	6,87	20,0	12,0	20,0	25,0	21,0	25,0
0,60	4,35	0,48	30,0	30,0	30,0	50,0	50,0	50,0
0,00	0,00	1,42	26,0	35,0	35,0	21,0	50,0	50,0
0,30	3,15	0,00	28,0	24,0	28,0	50,0	41,0	50,0
0,20	2,76	2,75	9999,0	9999,0	9999,0	9999,0	9999,0	9999,0
0,00	0,00	3,87	15,0	31,0	31,0	30,0	30,0	30,0
9999,00	9999,00	9999,00	28,0	34,0	34,0	20,0	22,0	22,0
0,00	0,00	3,46	18,0	12,0	18,0	20,0	16,0	20,0
2,50	0,00	1,05	50,0	45,0	50,0	50,0	50,0	50,0
1,40	0,00	0,00	14,0	21,0	21,0	20,0	19,0	20,0
9999,00	9999,00	9999,00	18,0	31,0	31,0	22,0	27,0	27,0
1,30	0,00	18,88	11,0	18,0	18,0	20,0	15,0	20,0
3,00	2,76	0,00	50,0	50,0	50,0	50,0	50,0	50,0
0,30	0,00	5,81	31,0	44,0	44,0	40,0	42,0	42,0
2,20	8,18	0,00	10,0	9,0	10,0	10,0	10,0	10,0
1,30	14,78	0,00	46,0	48,0	48,0	50,0	50,0	50,0
1,70	0,00	0,00	14,0	23,0	23,0	32,0	38,0	38,0
0,30	6,09	0,00	40,0	50,0	50,0	40,0	35,0	40,0
1,40	0,08	4,35	18,0	19,0	19,0	17,0	21,0	21,0
1,70	0,00	0,00	45,0	37,0	45,0	50,0	48,0	50,0
9999,00	9999,00	9999,00	21,0	21,0	21,0	25,0	28,0	28,0
9999,00	9999,00	9999,00	30,0	25,0	30,0	50,0	24,0	50,0
9999,00	9999,00	9999,00	21,0	21,0	21,0	20,0	25,0	25,0
1,80	0,00	10,70	50,0	50,0	50,0	9999,0	9999,0	9999,0
1,50	0,00	5,75	11,0	10,0	11,0	18,0	19,0	19,0
2,10	12,23	0,14	18,0	11,0	18,0	28,0	18,0	28,0
2,30	2,87	2,08	22,0	24,0	24,0	32,0	16,0	32,0
0,80	0,00	0,00	10,0	7,0	10,0	25,0	15,0	25,0
1,80	0,00	7,65	18,0	18,0	18,0	21,0	20,0	21,0
0,60	0,00	4,35	28,0	18,0	28,0	38,0	40,0	40,0
0,60	0,00	0,00	42,0	50,0	50,0	50,0	50,0	50,0
0,90	0,00	0,00	21,0	31,0	31,0	28,0	26,0	28,0
0,00	0,00	0,34	23,0	22,0	23,0	25,0	9999,0	25,0
0,20	0,00	0,34	25,0	50,0	50,0	38,0	40,0	40,0
0,40	0,32	0,47	45,0	30,0	45,0	42,0	48,0	48,0
0,00	2,26	0,00	15,0	10,0	15,0	13,0	11,0	13,0
0,40	36,81	1,14	19,0	12,0	19,0	24,0	25,0	25,0
0,00	0,00	0,00	10,0	10,0	10,0	14,0	14,0	14,0
0,40	4,40	0,00	20,0	16,0	20,0	19,0	14,0	19,0
0,00	2,87	0,00	12,0	12,0	12,0	14,0	13,0	14,0
0,90	2,61	2,81	25,0	25,0	25,0	25,0	25,0	25,0
2,20	0,00	3,48	27,0	27,0	27,0	26,0	25,0	26,0
2,10	0,07	0,41	50,0	50,0	50,0	50,0	50,0	50,0
1,20	0,00	14,78	25,0	25,0	25,0	45,0	35,0	45,0
2,00	14,78	19,63	38,0	24,0	38,0	23,0	24,0	24,0

VPT95BVEt	VPT95BVEa	sumVPT95I	catVPT95B	DNSinstab(	DNSpijn0n	DNStintel0	DNSgevoel	ScoreDNSo
0	0	0	0	0	0	0	0	0
0	0	0	0	0	0	0	0	0
0	0	0	0	0	0	0	0	0
9999	9999	9999	9999	0	0	0	0	0
0	0	0	0	0	0	0	0	0
0	0	0	0	0	0	0	0	0
0	0	0	0	0	0	0	0	0
0	0	0	0	0	0	0	0	0
0	0	0	0	9999	9999	9999	9999	9999
0	0	0	0	0	0	0	0	0
0	0	0	0	0	0	0	0	0
0	0	0	0	0	0	0	0	0
0	0	0	0	0	0	0	0	0
0	0	0	0	0	0	0	0	0
0	0	0	0	0	0	0	0	0
0	0	0	0	0	0	0	0	0
0	0	0	0	0	0	0	0	0
0	0	0	0	0	0	0	0	0
0	0	0	0	9999	9999	9999	9999	9999
0	0	0	0	9999	9999	9999	9999	9999
0	0	0	0	0	0	0	0	0
0	0	0	0	0	0	0	0	0
0	0	0	0	9999	9999	9999	9999	9999
0	0	0	0	0	0	0	0	0
0	0	0	0	9999	9999	9999	9999	9999
0	0	0	0	0	0	0	0	0
0	0	0	0	0	0	0	0	0
0	0	0	0	0	0	0	0	0
0	0	0	0	9999	9999	9999	9999	9999
0	0	0	0	0	0	0	0	0
0	0	0	0	9999	9999	9999	9999	9999
0	0	0	0	9999	9999	9999	9999	9999
0	0	0	0	0	0	0	0	0
0	0	0	0	0	0	0	0	0
0	0	0	0	9999	9999	9999	9999	9999
1	0	1	1	1	1	1	1	4
0	1	1	1	0	0	0	0	0
0	0	0	0	1	0	1	0	2
1	1	2	1	0	0	0	0	0
0	0	0	0	1	1	1	0	3
1	1	2	1	0	0	0	0	0
9999	9999	9999	9999	1	1	1	1	4
1	1	2	1	9999	9999	9999	9999	9999
0	0	0	0	1	0	0	0	1
1	0	1	1	9999	9999	9999	9999	9999
1	1	2	1	0	0	0	0	0
0	1	1	1	1	1	1	1	4
1	0	1	1	0	0	0	0	0
1	1	2	1	9999	9999	9999	9999	9999

0	0	0	0	9999	9999	9999	9999	9999
1	1	2	1	1	1	1	0	3
1	1	2	1	1	0	0	1	2
0	0	0	0	0	0	0	0	0
0	0	0	0	1	0	0	0	1
1	9999	1	1	0	0	1	0	1
0	0	0	0	1	0	1	0	2
0	1	1	1	1	1	0	0	2
0	0	0	0	1	0	0	0	1
1	1	2	1	9999	9999	9999	9999	9999
0	0	0	0	0	0	0	0	0
1	1	2	1	0	1	1	0	2
1	1	2	1	1	1	0	0	2
0	1	1	1	0	0	0	0	0
0	0	0	0	1	0	1	0	2
1	1	2	1	0	0	0	0	0
9999	9999	9999	9999	1	0	0	0	1
9999	9999	9999	9999	0	0	0	0	0
9999	9999	9999	9999	0	0	1	0	1
9999	9999	9999	9999	1	1	0	1	3
9999	9999	9999	9999	1	0	0	0	1
9999	9999	9999	9999	1	0	0	0	1
9999	9999	9999	9999	0	0	0	0	0
1	1	2	1	1	1	1	0	3
1	1	2	1	0	0	0	0	0
0	0	0	0	0	1	0	1	1
0	1	1	1	0	0	0	0	0
9999	9999	9999	9999	1	0	0	0	1
1	1	2	1	0	0	0	0	0
0	0	0	0	9999	9999	9999	9999	9999
1	1	2	1	1	1	1	0	3
1	1	2	1	0	0	0	0	0
1	1	2	1	9999	9999	9999	9999	9999
0	0	0	0	0	0	0	0	0
0	0	0	0	1	0	0	0	1
0	0	0	0	1	0	0	0	1
0	0	0	0	0	1	1	0	2
1	9999	1	1	9999	9999	9999	9999	9999
0	1	1	1	1	0	0	1	2
1	1	2	1	1	1	1	0	3
0	0	0	0	0	0	0	0	0
1	1	2	1	0	0	0	0	0
1	1	2	1	0	0	0	0	0
1	0	1	1	0	0	0	0	0
1	1	2	1	9999	9999	9999	9999	9999
1	1	2	1	1	1	1	1	4
0	1	1	1	1	0	1	0	2
1	9999	1	1	9999	9999	9999	9999	9999
0	0	0	0	9999	9999	9999	9999	9999
0	0	0	0	0	0	0	0	0

0	0	0	0	0	0	0	0	0
0	0	0	0	0	1	1	0	2
0	1	1	1	0	0	0	0	0
0	1	1	1	1	1	1	0	3
0	1	1	1	1	0	0	0	1
9999	9999	9999	9999	1	1	1	0	3
0	0	0	0	0	0	0	0	0
0	0	0	0	9999	9999	9999	9999	9999
0	0	0	0	0	0	0	0	0
1	1	2	1	0	0	0	0	0
0	0	0	0	1	1	1	0	3
1	0	1	1	9999	9999	9999	9999	9999
0	0	0	0	0	0	0	0	0
1	1	2	1	0	0	0	0	0
1	1	2	1	1	1	1	1	4
0	0	0	0	0	0	0	0	0
1	1	2	1	0	0	0	0	0
0	1	1	1	0	1	1	1	3
1	1	2	1	0	1	1	0	2
0	0	0	0	0	0	0	0	0
1	1	2	1	0	0	0	0	0
0	0	0	0	9999	9999	9999	9999	9999
0	1	1	1	9999	9999	9999	9999	9999
0	0	0	0	9999	9999	9999	9999	9999
1	9999	1	1	0	0	0	0	0
0	0	0	0	0	0	0	0	0
0	0	0	0	0	0	0	0	0
0	1	1	1	0	0	0	0	0
0	0	0	0	1	0	0	0	1
0	0	0	0	0	0	0	0	0
0	0	0	0	1	0	0	0	1
1	1	2	1	1	1	1	1	4
1	0	1	1	1	1	1	1	4
0	0	0	0	0	0	0	0	0
1	0	1	1	0	0	0	0	0
1	1	2	1	1	0	0	0	1
0	0	0	0	1	0	1	0	2
0	0	0	0	0	0	0	0	0
0	0	0	0	0	0	0	0	0
0	0	0	0	0	0	0	0	0
0	0	0	0	0	0	0	0	0
1	0	1	1	0	0	0	0	0
1	0	1	1	1	0	0	0	1
1	1	2	1	0	0	0	0	0
0	0	0	0	0	0	0	0	0
1	0	1	1	0	0	0	0	0

catDNS	NPDNSVPT	HKK1kg	HKK2kg	HKK3kg	SPPB2m44	SPPBtande	SPPBsmals	SPPBsemita
0	0	17	14	12	3,00	2,00	2,00	2,00
0	0	20	20	18	3,00	2,00	2,00	2,00
0	0	10	10	12	2,00	2,00	2,00	2,00
0	0	6	5	6	1,00	1,00	2,00	2,00
0	0	22	26	26	4,00	2,00	2,00	2,00
0	0	10	11	12	4,00	1,00	2,00	2,00
0	0	9999	9999	9999	3,00	1,00	2,00	2,00
0	0	10	8	7	9999,00	9999,00	9999,00	9999,00
9999	0	9999	9999	9999	2,00	2,00	2,00	2,00
0	0	33	39	33	4,00	2,00	2,00	2,00
0	0	19	22	22	4,00	2,00	2,00	2,00
0	0	34	39	40	4,00	2,00	2,00	2,00
0	0	19	17	19	4,00	2,00	2,00	2,00
0	0	27	26	28	4,00	2,00	2,00	2,00
0	0	14	15	16	4,00	2,00	2,00	2,00
0	0	45	45	44	4,00	2,00	2,00	2,00
0	0	46	51	54	4,00	2,00	2,00	2,00
0	0	40	39	42	4,00	2,00	2,00	2,00
9999	0	13	14	11	4,00	2,00	2,00	2,00
9999	0	40	40	37	4,00	2,00	2,00	2,00
0	0	40	42	41	4,00	2,00	2,00	2,00
0	0	26	25	30	4,00	2,00	2,00	2,00
9999	0	36	42	45	4,00	2,00	2,00	2,00
0	0	42	47	46	4,00	2,00	2,00	2,00
9999	0	18	18	16	4,00	2,00	2,00	2,00
0	0	22	22	22	4,00	2,00	2,00	2,00
0	0	24	24	26	4,00	2,00	2,00	2,00
0	0	38	34	32	4,00	2,00	2,00	2,00
9999	0	20	20	18	4,00	2,00	2,00	2,00
0	0	20	16	16	4,00	2,00	2,00	2,00
9999	0	24	24	24	4,00	2,00	2,00	2,00
9999	0	24	28	30	4,00	2,00	2,00	2,00
0	0	20	16	16	4,00	2,00	2,00	2,00
0	0	24	24	26	4,00	2,00	2,00	2,00
9999	0	19	18	18	4,00	2,00	2,00	2,00
1	2	22	22	19	3,00	0,00	2,00	2,00
0	1	20	20	21	4,00	2,00	2,00	2,00
1	1	20	20	22	3,00	0,00	2,00	2,00
0	1	14	14	14	2,00	2,00	2,00	2,00
1	1	10	11	10	1,00	0,00	2,00	2,00
0	1	9999	8	5	1,00	1,00	2,00	2,00
1	1	35	39	36	3,00	0,00	1,00	0,00
9999	1	14	9	14	4,00	2,00	2,00	2,00
1	1	24	22	22	3,00	2,00	2,00	2,00
9999	1	10	10	10	3,00	0,00	2,00	2,00
0	1	16	16	14	3,00	2,00	2,00	2,00
1	2	5	9999	9999	2,00	2,00	2,00	2,00
0	1	9	14	15	4,00	2,00	2,00	2,00
9999	1	6	7	6	2,00	9999,00	2,00	9999,00

9999	0	15	12	16	2,00	0,00	1,00	0,00
1	2	20	20	23	4,00	1,00	2,00	2,00
1	2	19	22	21	4,00	2,00	2,00	2,00
0	0	16	12	14	2,00	2,00	2,00	2,00
1	1	12	9	8	9999,00	0,00	0,00	0,00
1	2	26	30	30	4,00	2,00	2,00	2,00
1	1	9999	9999	9999	2,00	0,00	2,00	0,00
1	2	10	9999	9999	1,00	0,00	0,00	0,00
1	1	9999	9999	9999	1,00	1,00	2,00	2,00
9999	1	9999	9999	8	1,00	0,00	0,00	0,00
0	0	20	22	24	2,00	2,00	2,00	2,00
1	2	8	10	8	2,00	2,00	2,00	2,00
1	2	10	12	12	2,00	0,00	0,00	0,00
0	1	6	6	8	1,00	0,00	2,00	2,00
1	1	6	6	8	1,00	0,00	2,00	2,00
0	1	9999	9999	9999	1,00	1,00	2,00	2,00
1	1	28	27	27	3,00	9999,00	2,00	2,00
0	0	24	25	21	3,00	0,00	2,00	2,00
1	1	18	18	15	3,00	1,00	2,00	2,00
1	1	16	16	16	1,00	0,00	2,00	0,00
1	1	9999	9999	9999	3,00	9999,00	2,00	2,00
1	1	13	13	15	3,00	9999,00	2,00	2,00
0	0	9999	5	5	1,00	9999,00	2,00	2,00
1	2	10	11	10	2,00	1,00	2,00	2,00
0	1	16	18	16	1,00	0,00	2,00	2,00
1	1	9999	6	6	1,00	0,00	2,00	2,00
0	1	10	8	8	1,00	9999,00	2,00	2,00
1	1	9999	9999	9999	1,00	1,00	2,00	2,00
0	1	16	18	18	2,00	2,00	2,00	2,00
9999	0	9999	9999	9999	1,00	0,00	2,00	2,00
1	2	10	10	10	3,00	0,00	2,00	0,00
0	1	18	20	18	2,00	0,00	2,00	2,00
9999	1	9999	8	8	1,00	1,00	2,00	2,00
0	0	9999	9999	9999	1,00	0,00	0,00	0,00
1	1	18	17	16	2,00	1,00	2,00	2,00
1	1	8	7	9999	2,00	2,00	2,00	2,00
1	1	9999	9999	9999	3,00	1,00	2,00	2,00
9999	1	14	18	16	3,00	0,00	0,00	0,00
1	2	19	22	21	3,00	2,00	2,00	2,00
1	2	12	10	10	1,00	1,00	2,00	2,00
0	0	20	21	25	4,00	9999,00	2,00	2,00
0	1	16	17	19	2,00	1,00	2,00	2,00
0	1	36	36	36	3,00	2,00	2,00	2,00
0	1	34	35	35	3,00	1,00	2,00	2,00
9999	1	25	28	27	4,00	1,00	2,00	2,00
1	2	5	6	5	1,00	9999,00	2,00	2,00
1	2	5	9999	9999	3,00	0,00	2,00	0,00
9999	1	6	5	6	1,00	9999,00	2,00	2,00
9999	0	23	23	22	2,00	1,00	2,00	2,00
0	0	6	7	6	4,00	1,00	2,00	2,00

0	0	38	35	36	4,00	2,00	2,00	2,00
1	1	16	16	18	4,00	2,00	2,00	2,00
0	1	10	10	11	2,00	2,00	2,00	2,00
1	2	10	15	11	9999,00	0,00	0,00	0,00
1	2	16	16	16	3,00	1,00	2,00	2,00
1	1	8	6	7	2,00	9999,00	2,00	2,00
0	0	16	17	17	2,00	0,00	2,00	2,00
9999	0	8	6	7	1,00	2,00	2,00	2,00
0	0	9999	9999	5	1,00	0,00	1,00	1,00
0	1	18	18	20	4,00	1,00	2,00	2,00
1	1	20	19	16	4,00	2,00	2,00	2,00
9999	1	11	14	18	4,00	2,00	2,00	2,00
0	0	28	29	34	4,00	2,00	2,00	2,00
0	1	26	26	31	4,00	2,00	2,00	2,00
1	2	24	21	23	4,00	2,00	2,00	2,00
0	0	18	17	16	4,00	2,00	2,00	2,00
0	1	24	24	24	4,00	1,00	2,00	2,00
1	2	8	10	10	3,00	2,00	2,00	2,00
1	2	9	10	11	2,00	2,00	2,00	2,00
0	0	17	20	19	3,00	2,00	2,00	2,00
0	1	16	16	18	3,00	2,00	2,00	2,00
9999	0	23	20	22	4,00	2,00	2,00	2,00
9999	1	11	14	14	4,00	1,00	2,00	2,00
9999	0	7	9	8	1,00	1,00	2,00	2,00
0	1	13	13	10	4,00	2,00	2,00	2,00
0	0	16	14	18	4,00	2,00	2,00	2,00
0	0	38	39	42	4,00	2,00	2,00	2,00
0	1	24	23	20	4,00	1,00	2,00	2,00
1	1	14	10	14	4,00	1,00	2,00	1,00
0	0	22	21	25	9999,00	2,00	2,00	2,00
1	1	28	28	30	3,00	2,00	2,00	2,00
1	2	10	14	12	1,00	2,00	2,00	2,00
1	2	20	18	20	4,00	1,00	2,00	2,00
0	0	17	18	16	2,00	1,00	2,00	2,00
0	1	15	16	16	1,00	0,00	2,00	1,00
1	2	10	12	12	9999,00	0,00	2,00	2,00
1	1	10	12	13	1,00	0,00	2,00	2,00
0	0	26	24	24	2,00	2,00	2,00	2,00
0	0	12	12	12	1,00	1,00	2,00	2,00
0	0	8	8	8	1,00	1,00	2,00	2,00
0	0	24	22	25	2,00	1,00	2,00	2,00
0	1	34	36	33	4,00	2,00	2,00	2,00
1	2	48	48	48	4,00	2,00	2,00	2,00
0	1	26	25	25	4,00	2,00	2,00	2,00
0	0	36	36	36	4,00	2,00	2,00	2,00
0	1	48	48	50	4,00	2,00	2,00	2,00

SPPBbalanc	SPPBTCRsc	somSPPBsc	MPExp1	MPExp2	MPExp3	MPInsp1	MPInsp2	MPInsp3
4,00	0,00	7,00	40,87	54,95	9999,00	21,33	31,67	9999,00
4,00	3,00	10,00	84,18	85,16	9999,00	45,57	58,52	9999,00
4,00	0,00	6,00	45,39	80,40	69,39	29,59	41,76	36,42
3,00	0,00	4,00	30,00	21,45	33,30	14,14	15,19	20,02
4,00	0,00	8,00	115,26	127,55	113,41	50,08	55,57	40,24
3,00	1,00	8,00	62,00	72,57	52,61	23,85	31,92	26,95
3,00	0,00	6,00	50,25	38,22	29,33	18,64	7,49	9999,00
9999,00	9999,00	9999,00	94,28	75,54	61,77	16,48	14,94	14,24
4,00	0,00	6,00	9999,00	9999,00	9999,00	9999,00	9999,00	9999,00
4,00	4,00	12,00	94,75	133,30	115,45	64,87	82,10	96,51
4,00	2,00	10,00	54,42	58,56	70,34	20,24	37,22	26,05
4,00	4,00	12,00	93,31	118,59	89,40	47,90	33,21	55,52
4,00	1,00	9,00	9999,00	9999,00	9999,00	9999,00	9999,00	9999,00
4,00	3,00	11,00	87,88	98,37	105,69	60,75	78,97	70,93
4,00	3,00	11,00	75,60	74,02	71,48	73,48	82,66	72,96
4,00	4,00	12,00	171,00	143,00	146,00	103,77	111,24	113,35
4,00	3,00	11,00	124,89	108,54	115,69	116,72	124,35	129,51
4,00	3,00	11,00	96,27	89,75	77,07	39,77	9999,00	9999,00
4,00	2,00	10,00	94,97	115,36	123,40	50,45	68,42	66,41
4,00	4,00	12,00	111,34	108,42	9999,00	69,50	62,60	9999,00
4,00	4,00	12,00	112,33	114,04	113,72	78,40	88,76	82,51
4,00	4,00	12,00	90,68	119,65	107,37	99,13	120,76	118,32
4,00	3,00	11,00	9999,00	9999,00	9999,00	9999,00	9999,00	9999,00
4,00	4,00	12,00	96,52	90,79	92,78	52,06	67,96	59,39
4,00	1,00	9,00	55,41	63,97	60,68	45,24	49,77	47,80
4,00	4,00	12,00	97,01	95,87	93,07	57,64	50,86	55,25
4,00	2,00	10,00	86,42	90,81	97,03	49,71	46,03	45,38
4,00	4,00	12,00	92,77	97,52	99,40	61,32	49,68	54,20
4,00	1,00	9,00	87,16	90,39	73,15	34,09	37,73	35,21
4,00	3,00	11,00	97,23	122,44	9999,00	87,85	91,24	9999,00
4,00	4,00	12,00	89,47	85,87	80,51	58,78	72,09	70,96
4,00	3,00	11,00	154,97	136,68	9999,00	73,51	68,69	9999,00
4,00	4,00	12,00	130,91	120,30	114,59	89,92	9999,00	9999,00
4,00	4,00	12,00	110,37	106,00	98,85	72,44	69,21	66,32
4,00	3,00	11,00	101,28	103,31	9999,00	78,12	84,80	9999,00
2,00	1,00	6,00	9999,00	9999,00	9999,00	9999,00	9999,00	9999,00
4,00	1,00	9,00	64,12	78,19	83,64	56,54	58,47	33,29
2,00	1,00	6,00	9999,00	9999,00	9999,00	9999,00	9999,00	9999,00
4,00	0,00	6,00	67,89	65,72	73,01	27,19	26,29	24,39
2,00	0,00	3,00	55,97	55,45	46,85	26,29	26,50	11,94
3,00	0,00	4,00	41,06	49,53	38,10	41,74	32,61	34,12
0,00	0,00	3,00	98,50	117,25	111,34	56,30	75,49	55,85
4,00	2,00	10,00	91,23	89,63	101,79	65,30	60,03	87,18
4,00	0,00	7,00	91,63	92,08	98,02	31,94	36,85	34,97
2,00	0,00	5,00	23,74	26,56	13,10	11,81	7,52	16,84
4,00	0,00	7,00	61,11	64,38	57,77	39,30	35,38	29,99
4,00	0,00	6,00	79,05	90,02	97,21	48,85	47,95	28,90
4,00	1,00	9,00	67,07	79,26	81,27	14,13	16,08	16,42
9999,00	9999,00	9999,00	42,74	46,42	43,69	20,54	19,43	17,33

0,00	0,00	2,00	42,74	46,42	43,69	20,54	19,43	17,33
2,00	1,00	7,00	112,41	106,16	116,74	44,73	66,79	87,57
4,00	2,00	10,00	9999,00	9999,00	9999,00	9999,00	9999,00	9999,00
4,00	1,00	7,00	39,10	49,86	44,24	38,31	38,66	37,00
0,00	0,00	9999,00	41,21	28,92	37,36	22,74	27,08	28,87
4,00	1,00	9,00	9999,00	9999,00	9999,00	9999,00	9999,00	9999,00
0,00	0,00	2,00	9999,00	9999,00	9999,00	9999,00	9999,00	9999,00
0,00	0,00	1,00	11,38	29,01	12,83	9,66	3,41	9999,00
3,00	0,00	4,00	33,34	38,26	30,87	22,87	11,21	16,29
0,00	0,00	1,00	9999,00	9999,00	9999,00	9999,00	9999,00	9999,00
4,00	1,00	7,00	38,63	36,65	27,13	16,39	19,76	9,32
4,00	0,00	6,00	73,35	36,87	66,03	14,18	6,78	9999,00
0,00	0,00	2,00	57,46	50,14	55,97	44,98	35,94	33,85
2,00	0,00	3,00	53,21	56,79	78,35	8,45	16,42	16,51
2,00	0,00	3,00	32,31	42,79	48,18	32,64	41,83	28,71
3,00	0,00	4,00	36,44	25,39	29,92	9,24	18,69	23,28
9999,00	0,00	9999,00	52,43	58,42	57,64	24,97	27,08	28,74
2,00	0,00	5,00	97,53	110,31	103,29	37,88	42,33	35,88
3,00	0,00	6,00	78,05	77,58	77,37	20,50	32,99	29,03
1,00	0,00	2,00	46,83	49,57	44,58	11,96	15,07	14,24
9999,00	0,00	9999,00	31,68	31,34	33,15	7,19	6,53	6,50
9999,00	0,00	9999,00	30,68	32,45	35,21	44,55	44,96	37,28
9999,00	0,00	9999,00	43,50	47,90	9999,00	8,12	9999,00	9999,00
3,00	2,00	7,00	41,48	92,50	9999,00	31,97	31,58	9999,00
2,00	0,00	3,00	68,41	51,34	63,51	32,11	19,98	29,82
2,00	0,00	3,00	49,70	30,34	9999,00	12,65	2,71	9999,00
9999,00	0,00	9999,00	35,34	22,48	9999,00	22,29	12,20	9999,00
2,00	0,00	3,00	31,98	22,62	9999,00	9,59	11,62	9999,00
4,00	0,00	6,00	55,12	36,26	41,75	57,24	31,77	23,26
2,00	0,00	3,00	44,86	38,05	38,25	22,60	26,61	20,78
1,00	0,00	4,00	35,38	33,69	32,61	8,37	7,01	11,99
2,00	0,00	4,00	52,23	47,33	48,80	8,28	21,19	12,10
2,00	0,00	3,00	75,42	61,31	65,45	14,61	7,09	25,09
0,00	0,00	1,00	30,34	23,78	10,39	2,71	8,55	11,19
2,00	0,00	4,00	65,51	78,20	91,58	67,11	76,96	73,57
4,00	0,00	6,00	65,45	53,54	62,97	25,09	16,51	25,95
2,00	0,00	5,00	103,50	99,81	9999,00	51,70	55,72	9999,00
0,00	0,00	3,00	73,14	79,55	59,32	13,07	15,28	15,22
4,00	1,00	8,00	80,12	82,71	84,17	39,34	35,00	83,00
3,00	3,00	7,00	78,99	98,47	95,33	43,87	42,99	23,24
9999,00	0,00	9999,00	9999,00	9999,00	9999,00	9999,00	9999,00	9999,00
3,00	1,00	6,00	110,94	105,95	101,70	64,34	86,90	79,73
4,00	4,00	11,00	99,37	98,22	9999,00	73,03	52,94	9999,00
3,00	4,00	10,00	100,60	103,89	101,49	43,79	50,55	53,00
4,00	1,00	9,00	85,34	96,09	101,25	41,18	53,17	54,24
9999,00	0,00	9999,00	63,13	51,26	61,07	15,64	23,29	16,24
1,00	0,00	4,00	34,14	23,01	27,96	15,44	17,20	17,53
9999,00	0,00	9999,00	36,35	39,09	42,03	15,26	9,02	15,07
3,00	0,00	5,00	93,56	96,80	97,51	48,89	47,85	36,22
2,00	0,00	6,00	62,15	66,31	59,47	21,54	30,48	25,97

4,00	3,00	11,00	131,30	131,19	129,44	105,64	112,13	105,60
4,00	3,00	11,00	159,45	115,98	131,59	69,08	58,63	50,71
4,00	1,00	7,00	56,32	54,57	56,61	46,81	55,58	46,52
0,00	0,00	9999,00	26,59	20,80	9999,00	6,91	8,76	9999,00
3,00	1,00	7,00	68,43	73,17	86,22	58,58	67,42	68,03
9999,00	0,00	9999,00	9999,00	9999,00	9999,00	9999,00	9999,00	9999,00
2,00	0,00	4,00	48,53	58,60	9999,00	16,89	47,91	9999,00
4,00	0,00	5,00	69,92	38,85	48,53	13,07	12,22	16,89
0,00	0,00	1,00	48,69	63,95	63,61	39,68	30,23	9999,00
3,00	3,00	10,00	90,67	94,17	9999,00	41,88	38,23	9999,00
4,00	3,00	11,00	60,98	118,88	79,09	26,73	15,28	41,08
4,00	2,00	10,00	75,78	77,89	9999,00	32,34	37,98	9999,00
4,00	2,00	10,00	114,35	111,89	118,84	72,05	65,48	59,16
4,00	2,00	10,00	80,91	91,18	87,25	46,13	45,03	59,25
4,00	3,00	11,00	49,39	60,73	64,83	52,54	57,54	56,53
4,00	1,00	9,00	101,53	105,95	100,98	84,61	86,35	85,71
3,00	2,00	9,00	67,69	78,04	81,08	87,60	97,84	70,56
4,00	1,00	8,00	41,82	45,58	9999,00	18,10	23,70	9999,00
4,00	0,00	6,00	82,56	81,04	81,85	22,79	21,35	23,67
4,00	2,00	9,00	101,71	97,41	101,71	50,12	71,35	41,80
4,00	3,00	10,00	110,05	91,90	89,93	27,85	14,75	14,90
4,00	2,00	10,00	98,75	91,34	9999,00	48,86	55,42	9999,00
3,00	0,00	7,00	77,04	90,25	79,41	23,72	22,29	26,78
2,00	0,00	3,00	53,71	48,57	9999,00	31,74	16,06	9999,00
4,00	2,00	10,00	9999,00	9999,00	9999,00	9999,00	9999,00	9999,00
4,00	1,00	9,00	63,28	61,75	55,77	38,00	16,95	32,63
4,00	2,00	10,00	70,57	93,14	90,58	22,53	35,13	26,00
3,00	1,00	8,00	39,69	33,89	45,78	42,46	45,18	39,59
1,00	4,00	9,00	36,45	9999,00	9999,00	28,82	9999,00	9999,00
4,00	3,00	9999,00	90,64	97,41	94,24	61,62	65,70	59,45
4,00	4,00	11,00	44,22	75,17	85,27	45,03	70,59	81,10
4,00	1,00	6,00	20,04	27,51	26,18	14,84	13,64	12,76
3,00	1,00	8,00	61,55	59,89	64,79	42,58	48,88	53,28
3,00	3,00	8,00	48,00	54,56	18,96	32,66	23,77	7,78
1,00	0,00	2,00	3,72	12,03	10,98	14,19	9,32	13,27
2,00	9999,00	9999,00	18,96	9999,00	9999,00	7,78	9999,00	9999,00
2,00	1,00	4,00	33,31	25,70	25,73	5,81	23,06	6,78
4,00	1,00	7,00	46,17	48,82	40,30	5,83	9,07	13,04
3,00	1,00	5,00	48,72	46,33	32,30	26,66	27,60	46,42
2,00	1,00	4,00	9999,00	47,82	31,44	40,05	22,62	16,82
2,00	3,00	7,00	9999,00	9999,00	9999,00	9999,00	9999,00	9999,00
4,00	4,00	12,00	9,53	8,80	12,31	5,97	9,69	11,07
4,00	4,00	12,00	38,12	11,65	26,08	13,92	13,42	14,89
4,00	3,00	11,00	2,62	4,87	2,55	3,31	4,32	5,67
4,00	4,00	12,00	8,91	17,38	8,48	4,41	5,49	6,19
4,00	3,00	11,00	6,50	17,93	18,98	6,01	6,27	6,85

piekflow1	piekflow2	piekflow3	filter_$
400,00	410,00	460,00	1
350,00	400,00	400,00	1
480,00	290,00	380,00	1
320,00	370,00	310,00	1
360,00	440,00	450,00	1
150,00	200,00	250,00	1
150,00	250,00	200,00	1
170,00	260,00	250,00	1
150,00	190,00	140,00	1
550,00	550,00	550,00	1
540,00	550,00	530,00	1
690,00	680,00	690,00	1
9999,00	9999,00	9999,00	1
490,00	490,00	490,00	1
380,00	410,00	390,00	1
630,00	590,00	610,00	1
720,00	720,00	720,00	1
650,00	600,00	600,00	1
430,00	500,00	470,00	1
520,00	500,00	480,00	1
660,00	660,00	650,00	1
670,00	660,00	660,00	1
510,00	490,00	500,00	1
550,00	540,00	550,00	1
390,00	390,00	370,00	1
380,00	430,00	430,00	1
350,00	380,00	240,00	1
530,00	540,00	600,00	1
300,00	320,00	350,00	1
480,00	470,00	450,00	1
490,00	480,00	500,00	1
450,00	400,00	370,00	1
460,00	480,00	480,00	1
330,00	340,00	330,00	1
320,00	270,00	410,00	1
9999,00	9999,00	9999,00	0
350,00	380,00	340,00	0
9999,00	9999,00	9999,00	0
160,00	300,00	320,00	0
150,00	150,00	120,00	0
200,00	200,00	150,00	0
480,00	370,00	350,00	0
350,00	370,00	370,00	0
400,00	270,00	300,00	0
80,00	100,00	100,00	0
240,00	200,00	230,00	0
250,00	270,00	260,00	0
350,00	250,00	230,00	0
320,00	200,00	250,00	0

320,00	200,00	250,00	0
300,00	340,00	300,00	0
340,00	400,00	400,00	0
150,00	160,00	180,00	0
60,00	80,00	90,00	0
9999,00	9999,00	9999,00	0
9999,00	9999,00	9999,00	0
9999,00	9999,00	9999,00	0
170,00	170,00	150,00	0
200,00	185,00	200,00	0
210,00	220,00	230,00	0
80,00	100,00	100,00	0
120,00	200,00	210,00	0
200,00	250,00	280,00	0
200,00	210,00	220,00	0
100,00	140,00	200,00	0
250,00	280,00	270,00	0
400,00	490,00	410,00	0
360,00	270,00	300,00	0
130,00	120,00	80,00	0
120,00	60,00	70,00	0
100,00	120,00	170,00	0
100,00	150,00	160,00	0
170,00	190,00	200,00	0
120,00	220,00	170,00	0
60,00	200,00	160,00	0
150,00	100,00	180,00	0
70,00	60,00	60,00	0
140,00	260,00	290,00	0
40,00	70,00	80,00	0
150,00	250,00	220,00	0
200,00	230,00	350,00	0
120,00	160,00	120,00	0
150,00	100,00	130,00	0
460,00	460,00	470,00	0
90,00	80,00	70,00	0
220,00	270,00	300,00	0
250,00	270,00	260,00	0
360,00	390,00	410,00	0
110,00	200,00	300,00	0
9999,00	9999,00	9999,00	0
450,00	460,00	470,00	0
470,00	500,00	490,00	0
270,00	370,00	350,00	0
310,00	370,00	430,00	0
200,00	250,00	230,00	0
70,00	70,00	120,00	0
150,00	200,00	180,00	0
440,00	470,00	470,00	0
270,00	270,00	200,00	0

440,00	400,00	440,00	0
470,00	500,00	470,00	0
150,00	130,00	250,00	0
9999,00	9999,00	9999,00	0
280,00	370,00	370,00	0
9999,00	9999,00	9999,00	0
350,00	600,00	350,00	0
300,00	250,00	250,00	0
100,00	130,00	130,00	0
230,00	160,00	230,00	0
400,00	400,00	400,00	0
350,00	360,00	360,00	0
580,00	570,00	510,00	0
200,00	270,00	270,00	0
290,00	330,00	300,00	0
410,00	450,00	450,00	0
520,00	540,00	550,00	0
160,00	160,00	230,00	0
220,00	250,00	350,00	0
450,00	470,00	490,00	0
330,00	350,00	380,00	0
370,00	390,00	370,00	0
350,00	420,00	390,00	0
250,00	350,00	320,00	0
320,00	320,00	300,00	0
260,00	300,00	360,00	0
550,00	540,00	560,00	0
160,00	210,00	330,00	0
210,00	240,00	280,00	0
450,00	450,00	460,00	0
380,00	385,00	490,00	0
380,00	380,00	240,00	0
180,00	250,00	250,00	0
140,00	180,00	180,00	0
90,00	100,00	110,00	0
150,00	100,00	100,00	0
130,00	160,00	130,00	0
320,00	310,00	310,00	0
224,00	200,00	210,00	0
9999,00	9999,00	9999,00	0
150,00	150,00	150,00	0
575,00	500,00	500,00	0
360,00	530,00	560,00	0
100,00	140,00	130,00	0
220,00	270,00	170,00	0
550,00	575,00	600,00	0
